# IFNβ1 secreted by breast cancer cells undergoing chemotherapy reprograms stromal fibroblasts to support tumour growth after treatment

**DOI:** 10.1002/1878-0261.12905

**Published:** 2021-02-11

**Authors:** Ana Maia, Zuguang Gu, André Koch, Mireia Berdiel‐Acer, Rainer Will, Matthias Schlesner, Stefan Wiemann

**Affiliations:** ^1^ Division of Molecular Genome Analysis German Cancer Research Center (DKFZ) Heidelberg Germany; ^2^ Faculty of Biosciences University of Heidelberg Germany; ^3^ Computational Oncology Molecular Diagnostics Program National Center for Tumour Diseases (NCT) and German Cancer Research Center (DKFZ) Heidelberg Germany; ^4^ DKFZ‐HIPO (Heidelberg Center for Personalized Oncology) Germany; ^5^ Department of Women's Health Tübingen Eberhard‐Karls‐University Tübingen Germany; ^6^ Genomics and Proteomics Core Facility German Cancer Research Center (DKFZ) Heidelberg Germany; ^7^ Bioinformatics and Omics Data Analytics German Cancer Research Center (DKFZ) Heidelberg Germany

**Keywords:** breast cancer, chemotherapy, fibroblasts, IFNβ1, tumour microenvironment

## Abstract

Chemotherapy (CTX) remains the standard of care for most aggressive tumours, including breast cancer (BC). In BC chemotherapeutic regimens, the maximum tolerated dose of cytotoxic drugs is administered at regular intervals, and cancer cells can re‐grow or adapt during the resting periods between cycles. The impact of the tumour microenvironment on the fate of cancer cells after CTX remains poorly understood. Here, we show that paracrine signalling from CTX‐treated cancer cells to stromal fibroblasts can drive cancer cell recovery after cytotoxic drug withdrawal. Interferon β1 (IFNβ1) secreted by cancer cells following treatment with high doses of CTX instigates the acquisition of an anti‐viral state in stromal fibroblasts. This state is associated with an expression pattern here referred to as interferon signature (IFNS), which encompasses several interferon‐stimulated genes (ISGs), including numerous pro‐inflammatory cytokine genes. This crosstalk is an important driver of the expansion of BC cells after CTX, and IFNβ1 blockade in tumour cells abrogated their fibroblast‐dependent recovery potential. Analysis of human breast carcinomas supported a link between CTX‐induced IFNS in tumour stroma and poor response to CTX treatment. First, IFNβ1 expression in human breast carcinomas was found to inversely correlate with recurrence free survival (RFS). Second, using laser capture microdissection data sets, we show a higher expression of IFNS in the stromal tumour compartment compared to the epithelial one and this signature was found to be more prominent in more aggressive subtypes of BC (basal‐like), pointing to a pro‐tumorigenic role of this signature. Moreover, IFNS was associated with higher recurrence rates and a worse outcome in BC patients. Our study unravels a novel form of paracrine communication between cancer cells and fibroblasts that ultimately results in CTX resistance. Targeting this axis has the potential to improve CTX outcomes in patients with BC.

## Graphical abstract

Treatment with high doses of chemotherapy leads to the secretion of IFNβ1 into the tumour microenvironment. Exposure of stromal fibroblasts to IFNβ1 induces an anti‐viral, pro‐inflammatory signature associated with the expression of several interferon stimulated genes. This reprogramming drives a pro‐tumorigenic state in fibroblasts that facilitates cancer cell recovery after treatment and promotes cancer relapse in patients with breast cancer.

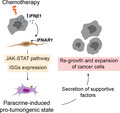

**Abbreviations**
BC: breast cancer
BUB1: budding uninhibited by benzimidazoles 1
CAF: cancer‐associated fibroblast
CC: co‐culture
CCL5: C‐C motif chemokine ligand 5
CDT1: chromatin licensing and DNA replication factor 1
CTX: chemotherapy
CXCL10: C‐X‐C motif chemokine ligand 10
DAMPs: damage‐associated molecular patterns
DAPI: 4′,6‐diamidino‐2‐phenylindole
DDX58: DExD/H‐Box Helicase 58
EdU: 5‐ethynyl‐2′‐deoxyuridine
FUCCI: fluorescent ubiquitination‐based cell cycle indicator
GO: gene ontology
GSEA: gene set enrichment analysis
IFIH1: interferon Induced with Helicase C Domain 1
IFN: interferon
IFNA: interferon alpha
IFNAR1: interferon alpha receptor 1
IFNB1: interferon beta 1
IFNG: interferon gamma
IFNL: interferon lambda
IFNLR1: interferon lambda receptor 1
IFNS: interferon signature
IL10RB: interleukin 10 receptor subunit beta
IL28RB: interleukin 28 receptor subunit beta
IL6: interleukin 6
IRF: interferon regulatory factors
ISG: interferon‐stimulated genes
ISG15: interferon‐stimulated protein, 15 KDa
KD: knock‐down
MC: mono‐culture
NF: normal fibroblast
OAS1: 2'‐5'‐Oligoadenylate Synthetase 1
PAMPs: pathogen‐associated molecular patterns
PRRs: pattern recognition receptors
RFP: red fluorescent protein
RFS: recurrence free survival
RT‐qPCR: reverse transcription quantitative PCR
siRNA: short interference RNA
STAT: signal transducer and activator of transcription
TCS: tumour cell secretome
TTK: threonine tyrosine kinase

## 1 Introduction

Breast cancer (BC) is still the most common type of malignancy and is responsible for the highest number of cancer‐related deaths among women [1, 2]. For a large number of cancer patients, chemotherapy (CTX) is still the standard of care and despite the initial good response rates, patients often return to the clinic with relapses. Therapeutic failure often arises from the expansion of clones of resistant cancer cells. These cells can be present in the initial tumour mass, but they can also arise later through adaption. Nevertheless, nonresistant tumour cells that are not eliminated at the time of treatment also play an important role in the development of relapses. In fact, drug‐tolerant tumour cells that do not undergo apoptosis or senescence after treatment can persist in the patient body, adapt and later give rise to tumour recurrences [3]. In BC, CTX is still given as cycles of maximum tolerated doses, which allows cancer cells to re‐grow and adapt during the resting periods. How cancer cells survive and escape therapy and what role stromal cells play in this process is still unclear.

The tumour microenvironment is an important player during all steps of tumour development and progression, and in BC, the majority of the stroma is constituted by cancer‐associated fibroblasts (CAFs). CAFs originate from a variety of cell types but the most prominent source is resident fibroblasts that become activated and then display an enhanced secretory profile [4]. By secreting a variety of growth factors, cytokines and extracellular matrix, CAFs are known to exert their control over an array of processes, including resistance to CTX [5, 6]. On the other hand, the phenotype and activation profile of CAFs are equally influenced by their surroundings, especially, but not exclusively, by cancer cells [4]. In the context of CTX, it is increasingly evident that these agents strongly modulate the secretory profile of cancer cells [7]. Alterations in the array of secreted factors dictate the interaction between cancer and stromal cells. Comprehending how these cell types communicate in the context of CTX is essential to understand therapeutic outcome and may provide insights into promising new drug targets.

Type I interferons (IFN) belong to the cytokine family and are secreted signalling molecules that are involved in the response of the immune system to infections [8]. Recognition of pathogen‐associated molecular patterns by specialized receptors named pattern recognition receptors (PRRs) leads to the expression of type I IFN [9, 10]. It is also known that this signalling is not only activated by foreign organisms, but that PRRs can also recognize self‐derived danger signals named damage‐associated molecular patterns (DAMPs) [11]. Such danger molecules often derive from damaged or dying cells making tumours often DAMP‐rich environments. Cytoplasmic nucleic acid sensors that recognize these danger molecules and induce type I IFN expression have been broadly described to be strongly activated in tumours as a response to ionizing therapy [12, 13, 14, 15]. All type I IFNs, which comprise 13 IFNα isoforms and IFNβ1, bind a common receptor formed by the heterodimer of interferon alpha receptor 1 (IFNAR1) and IFNAR2. Binding of type I IFNs to its receptor triggers a signalling cascade that culminates with the activation of signal transducer and activator of transcription (STAT), which then mediates the transcription of ISGs [16, 17]. ISGs expression in cancer has been associated with a gene signature that predicts response to both radiation and CTX [18, 19]. Expression of these ISGs is often driven by an antiviral response in cancer cells and was later shown to be induced in a paracrine manner by fibroblasts in cancer cells to promote chemoresistance [5]. The role of IFN signalling in cancer and its impact in dictating response to CTX is still poorly understood with studies showing both tumour‐inhibitory [20, 21, 22] and tumour‐promoting [23, 24, 25] activities.

In this study, we set out to investigate the impact of stromal fibroblasts on the fate of cancer cells after exposure to high doses of cytotoxic drugs and unravel the communication axis between these two cell types in a CTX‐dependent context. We show that stromal fibroblasts strongly increase the re‐growth of cancer cells after treatment with high doses of drugs and identified IFNβ1 as an important factor of the therapy‐induced tumour cell secretome (TCS). Secretion of IFNβ1 by CTX‐treated cancer cells led to an antiviral response in stromal fibroblasts, associated with the expression of several ISGs such as DExD/H‐Box Helicase 58 (*DDX58*), interferon Induced with Helicase C Domain 1 (*IFIH1*), interferon‐stimulated protein, 15 KDa (*ISG15*) and 2'‐5'‐Oligoadenylate Synthetase 1 (*OAS1*). Ablation of cancer cell‐derived IFNβ1 resulted in the abrogation of this activation state in fibroblasts and more importantly decreased the recovery potential of cancer cells after CTX treatment. Thus, our study defined a novel pro‐tumorigenic state of fibroblasts that is dependent on type I IFN signalling and that drives the escape of cancer cells from therapy.

## 2 Materials and methods

### 2.1 Isolation and establishment of primary culture of human fibroblasts

Generation of primary cultures from breast fibroblasts was done in collaboration with the National Centre for Tumour diseases (NCT) in Heidelberg and the Women’s Clinic in Tübingen. The scientific use of human tissue samples was approved by the medical faculty of the University Hospital Tübingen (ethical vote: 150/2018BO2) and by the Medical Faculty of Heidelberg (ethical vote: S‐392/2015). Isolation of fibroblasts from fresh tissues was performed as previously reported [26]. Very briefly, tissue was digested in collagenase (Sigma‐Aldrich, Saint‐Louis, MO, USA) for 2 h and the single cell suspension obtained was seeded in a cell culture dish. Preferential attachment to plastic by fibroblasts resulted in a population of cells enriched for this cell type. Five fibroblast lines were isolated and established for this study. Two pairs of fibroblasts isolated from the same patient from nonmalignant [normal fibroblast (NF)] and tumour sites (CAF) were obtained. Nonmalignant fibroblasts were collected from a distal area of the tumour site within the same breast. Primary fibroblasts in experiments were used until they reached passage 8 or were in culture for a maximum of 1.5 months, after which they were discarded.

### 2.2 Cell culture

All BC cell lines were obtained from ATCC and regularly authenticated by multiplex cell line authentication (Multiplexion GmbH, Friedrichshafen, Germany) and tested for mycoplasma contamination by PCR. BC cell lines and primary human fibroblasts were grown in full growth media [Dulbecco's Modified Eagle's medium (DMEM) supplemented with 10% FCS, 1% penicillin/streptavidin and 1% glutamine]. MCF7‐fluorescent ubiquitination‐based cell cycle indicator (FUCCI) cells were generated by infection with lentivirus‐expressing mKO2‐hCdt1(30/120)/pCSII‐EF and mAG‐hGeminin(1/110)/pCSII‐EF. In all experiments, cells were kept in reduced serum media (DMEM supplemented with 2.5% FCS, 1% penicillin/streptavidin and 1% glutamine).

### 2.3 Lentiviral infection

For generation of lentiviral particles, HEK293FT cells (Thermo Fisher Scientific, Dreieich, Germany) were co‐transfected with either mKO2‐hCdt1(30/120)/pCSII‐EF or mAG‐hGeminin(1/110)/pCSII‐EF) and second‐generation viral packaging plasmids VSV.G (Addgene #14888) and psPAX2 (Addgene #12260, Watertown, MA, USA). Forty‐eight hours after transfection, virus containing supernatant was removed and cleared by centrifugation (5 min/500 g). The supernatant was passed through a 0.45‐μm filter to remove remaining cellular debris. MCF7 cells were transduced with lentiviral particles at 75% confluency in the presence of 10 μg·mL^−1^ polybrene (Merck, Darmstadt, Germany). Twenty‐four hours after transduction, virus containing medium was replaced with selection medium for the respective expression constructs to start the selection. After 2 weeks of selection, double positive cells for red fluorescent protein (RFP) and GFP were further enriched using FACS.

### 2.4 Drug response

In a black 96‐well plate (Greiner Bio‐one, Kremsmünster, Austria), 1500–2000 cells per well were plated (day 0). At day 1, reduced serum medium with different concentrations of epirubicin (Biomol GmbH, Hamburg, Germany) and paclitaxel (Biomol GmbH) were added for 3 days. At defined time points, cells were imaged and analysed using the ImageXpress Micro Confocal microscope (Molecular Devices, Sunnyvale, CA, USA).

### 2.5 Colony formation (recovery assays)

Cancer cells were seeded at a low density (5 × 10^4^ cells per well) in a six‐well companion plate (Corning, NY, USA). The next day, chemotherapeutic agents were diluted according to Table S1 in reduced serum media (DMEM supplemented with 2.5% FCS, 1% P/S and 1% glutamine) and added to the cancer cells for 3 days. 2 × 10^4^ primary fibroblasts were seeded in a 6.5‐mm trans‐well with 0.4‐µm pore (Corning). After the 3 days incubation time, cancer cells were washed three times with PBS and allowed to recovery in reduced serum media in the absence [mono‐culture (MC)] or presence of a trans‐well with fibroblasts [co‐culture (CC)]. Media was refreshed every 4–5 days. After a minimum recovery period of 15 days, cancer cells were fixed in methanol for 10 min at room temperature (RT) and stained with crystal violet for 1 h. Crystal violet was removed, the wells were washed with distilled water and allowed to dry overnight. Plates were then scanned using an EPSON Perfection scan and images were analysed using imagej (NIH, Bethesda, MD, USA).

### 2.6 Conditioned media

MCF7 (2 × 10^5^ cells per well) and HS578T (1.125 × 10^5^ cells per well) were seeded in 6‐well plates and treated with 4 and 8 nm paclitaxel, respectively. After 3 days of incubation with chemotherapeutic drugs, cells were washed and fresh reduced serum media was added. Seventy‐two hours conditioned media (CM) from cancer cells was collected and filtered with a 0.45‐µm filter (Millex, Merck Millipore, Darmstadt, Germany) before adding to fibroblasts. If additional treatment of CM was done, the supernatant was split into four conical tubes and incubated with either DNase I (Sigma‐Aldrich), RNase A (Qiagen, Hilden, Germany), at 95 ºC or just kept at RT. DNase I and RNase A incubation was done for 30–45 min at RT and CM was kept at 95 ºC for no longer than 5 min. Afterwards, the pretreated CM was added to fibroblasts that had previously been seeded in a six‐well plate at a confluency of 2 × 10^4^ cells. Fibroblasts were incubated for 2 days with CM, and their cell pellets were collected for gene expression analysis. For exosome depletion, cells were grown in DMEM supplemented with exosome‐depleted FCS. Seventy‐two hours CM from cancer cells was collected and ultracentrifuged for 2 h at 100 000 ***g***. Supernatant was collected to a new tube, and the exosome‐enriched pellet fraction was resuspended and both fractions were added to the fibroblasts.

### 2.7 Type I interferon blocking antibodies

For blocking antibody treatment, supernatant from paclitaxel‐treated cancer cells was collected and an anti‐interferon beta 1 (IFNB1; R&D, AF814‐SP) antibody at a concentration of 0.2 µg·mL^−1^ was added. Fibroblasts were cultured in the supernatant of cancer cells for 48 h before being collected for gene expression analyses.

### 2.8 Immunofluorescence

For the characterization of primary fibroblasts isolated from patients, cells were seeded in glass coverslips. The following day, cells were fixed with 4% paraformaldehyde for 10 min at room temperature, permeabilized with 0.125% Triton X‐100 for 10 min and blocked with 4% BSA for 1 h. Primary antibodies against Fibronectin (ab2413, 1 : 100; Abcam, Cambridge, UK) and Vimentin (sc‐6260, 1 : 50; Santa Cruz, CA, USA) were incubated overnight in a humidified chamber at 4 ºC. Coverslips were washed three times with PBS containing 0.1% Tween‐20 and incubated for 1 h at room temperature with the respective secondary antibody (1 : 400; Abcam). Finally, coverslips were again washed and mounted in ProLong Diamond Antifade mounting media containing 4′,6‐diamidino‐2‐phenylindole (DAPI; Thermo Fisher Scientific, Waltham, MA, USA). Samples were imaged with Zeiss Cell Observer inverted microscope and processed using imagej (NIH).

### 2.9 Flow cytometry

Analysis of expression of the activation marker αSMA was done in the fibroblast lines isolated from patient samples using flow cytometry. Primary fibroblasts were grown in reduced serum media for 3 days after which they were trypsinized and collected for antibody staining. Briefly, 1 × 10^4^ cells were washed one time with PBS and fixed with CytoFix/CytoPerm solution (BD Biosciences, Franklin Lakes, NJ, USA) for 10 min on ice. Afterwards, cells were washed one time with 500 µL of 1× Perm/Wash Buffer (BD Biosciences) diluted in PBS and incubated with 0.02 µg of αSMA (#50‐9760‐82, eBioscience, Thermo Fisher Scientific, San Diego, CA, USA) antibody for 30 min on ice. One more washing step with 1× Perm/Wash Buffer (BD Biosciences) was done, and cells were acquired in the flow cytometer (BD FACS Canto II). Results were analysed using BD FACS DIVA (BD Biosciences).

### 2.10 Live cell imaging

5 × 10^4^ MCF7‐FUCCI cells were seeded in a six‐well plate in full growth media. After cells attached to the well bottom, growth media was replaced by Leibovitz's L‐15 medium (Gibco, Thermo Fisher Scientific, Waltham, MA, USA) supplemented with 2.5% FCS and cells were imaged every 20 min overnight with the motorized widefield microscope Cell Observer (Zeiss, Oberkochen, Germany). Images were analysed and processed using imagej (NIH).

### 2.11 Apoptosis analysis

Cancer cells were seeded (5 x 10^4^ cells per well), treated with either epirubicin or paclitaxel for 3 days and allowed to recover in the absence of drugs either in MC or CC with fibroblasts. At selected time points, supernatant and attached cells were collected and stained with DAPI for 5 min before acquisition in BD FACS Canto II flow cytometer (BD Biosciences). Results were analysed using the BD FACS DIVA (BD Biosciences).

### 2.12 Cell cycle

Investigation of the cell cycle profile of cancer cells was done using 5‐ethynyl‐2’‐deoxyuridine (EdU) labelling and the FUCCI system. Cancer cells were seeded (5 × 10^4^ cells per well), treated with CTX for 3 days and allowed to recover in the absence of drugs either in MC or CC with fibroblasts. At selected time points, 10 µm of EdU was diluted in reduced serum media and added for 30 h to cultures. After the incubation time, cancer cells were fixed in 4% PFA for 20 min at RT and stained according to the Click‐iT EdU imaging kit protocol (Invitrogen, Waltham, MA, USA). Stained cancer cells were imaged and quantified using the ImageXpress Micro Confocal microscope (Molecular Devices, Sunnyvale, CA, USA). FUCCI system analysis was done using flow cytometry. Briefly, MCF7‐FUCCI cells were seeded and submitted to the same experimental setup described above. Cells were collected by trypsinization, and the channels RFP and GFP were acquired in BD FACS Canto II flow cytometer (BD Biosciences). Results were analysed using the BD FACS DIVA (BD Biosciences).

### 2.13 Senescence

Chemotherapy‐treated MCF7 in MC and CC with fibroblasts were fixed at different time points of recovery in 4% PFA for 10 min. Cells were stained for SA‐β‐galactosidase activity overnight using a Cellular Senescence Assay kit (Merck Millipore) and imaged the next day in the motorized widefield microscope Cell Observer (Zeiss). Images were analysed and quantified using imagej (NIH).

### 2.14 RNA sequencing

Total RNA was isolated from cells using the RNeasy Mini Kit (Qiagen) according to manufacturer’s protocol. RNA concentration and purity were analysed using NanoDrop 2000 spectrophotometer (Thermo Fisher Scientific). Samples were sequenced in the DKFZ Genomics and Proteomics Core Facility using Illumina platform HiSeq 4000 v4 single‐read 50bp. Reads were mapped to the human reference genome (build 37, version hs37d5) using STAR (version 2.5.2b) [27] and a 2‐pass alignment. Duplicate reads were marked with sambamba (version 0.6.5) [28] using 8 threads. Then, reads were sorted by position using SAMtools (version 1.6) [29]. BAM file indexes were generated using sambamba. Quality control analysis was performed using the SAMtools flagstat command, and the rnaseqc tool (version v1.1.8.1) with the 1000 Genomes assembly and gencode 19 gene models. FeatureCounts (version 1.5.1) [30] was used to perform gene specific read counting over exon features based on the gencode V19 gene model. The quality threshold was set to 255 (which indicates that STAR found a unique alignment). For total library abundance calculations, during FPKM/TPM expression values estimations, all genes on chromosomes X, Y, MT, and rRNA and tRNA were omitted, as they possibly introduce library size estimation biases. Volcano plots showing differentially expressed genes were created using the online tool VolcaNoseR [31].

### 2.15 Transfections

Transfections were done using Lipofectamine RNAimax (Invitrogen, California, USA) according to manufacturer’s instructions. short interference RNA (siRNAs; Dharmacon, Colorado, USA) were used at a final concentration of 20 nm and are shown in Table S2. Briefly, the day after cells were seeded, a mix containing the transfection reagents and respective siRNAs was added dropwise to the cells. Cells were incubated for 6 h with the transfection mix, after which the media was aspirated and fresh media was added.

### 2.16 RNA extraction

Total RNA was isolated from cells using the RNeasy Mini Kit (Qiagen) according to manufacturer’s protocol. RNA concentration and purity were analysed using NanoDrop 2000.

### 2.17 Reverse transcription quantitative PCR (RT‐qPCR)

cDNA synthesis was done using the RevertAid H Minus First Strand cDNA Synthesis Kit (Thermo Fisher Scientific). Primers for gene expression analysis were designed using the Roche UPL Design Center (Roche Applied Science, Penzberg, Germany) and are shown in Table S3. All experiments were performed in triplicates and relative quantification was done using the 2^−ΔCt^ method. Relative expression was normalized to the house‐keeping genes *ACTB* and *PUM1*.

### 2.18 Western Blot

Fibroblasts were harvested and stored at −80 °C. Cell pellets were lysed in Lysis Buffer containing 20 mm Tris/HCl (pH7.5), 150 mm NaCl, 1% Triton X‐100, 1 mm DTT, 2 mm EDTA, 1 mm NaF, 1 mm Ortho‐Vanadate, 25 mm beta‐glycerophosphate and protease inhibitor cocktail (Roche Applied Science). Protein concentration of the samples was measured with BCA protein assay kit (Thermo Fisher Scientific) and quantified with GloMax microplate reader (Promega GmbH, Walldorf, Germany). Samples were loaded in a polyacrylamide gel and separated by SDS/PAGE. Proteins were transferred from the polyacrylamide gel to a PVDF membrane (Merck Millipore). Membranes were blocked with Rockland blocking buffer (Biomol GmbH) for 1 h at RT and incubated with the primary antibodies (Table S4) overnight at 4 °C. After washing a minimum of three times for at least 15 min with 0.1% Tween in TBS (TBS‐T), the membrane was incubated with a fluorescently labelled secondary antibody (Goat anti‐Rabbit Alexa Fluor 680 #A32734 against STAT1; Goat anti‐Mouse Alexa Fluor 800 #A32730 against ACTB; Thermo Fisher Scientific) at RT for 1 h. Finally, the membranes were washed with TBS‐T and scanned with Odyssey infrared imaging system (LI‐COR Biosciences, NE, USA). Images were then processed using imagej (NIH).

### 2.19 ELISA

ELISA was used to quantify the amounts of IFNβ1 in the supernatant of cancer cells to validate the IFNβ1 silencing by the interference RNA. MCF7 was seeded in full growth media in a six‐well plate at a confluency of 2 × 10^5^. The next day, siIFNβ1 plus transfection mix was added to the cells and incubated for 6 h. Transfection media was aspirated and reduced serum media with 10 µg·mL^−1^ of Poly(I:C) (InvivoGen, CA, USA) was added to the cells for 3 days. Secreted IFNβ1 was detected with human IFN‐beta DuoSet ELISA kit (R&D systems, Minneapolis, MN, USA) according to manufacturer’s instructions. Optical density was measured at 450 nm using GloMax microplate reader (Promega GmbH). A standard curve was generated using the absorbance values of the standards and used to calculate the concentration of IFNβ1 in each sample.

### 2.20 Gene set enrichment analysis

Investigation of the signatures enriched in upregulated genes was done using the online tool MSigDB in which a list with the name of the identified genes was loaded and the collection of interest was selected. For the generation of normalized enrichment scores (NES), a list containing all genes and their relative fold change between the conditions in analysis was generated and used to carried out the gene set enrichment analysis (GSEA) using GSEA v.4.0.3 [32, 33]. Comparison between groups (CAF1_CC‐U vs CAF1_CC‐P; MCF7_MC‐U vs MCF7_MC‐E/P) was done using the gene sets indicated in each figure. Gene sets were extracted from GSEA and the gene list for each signature is publicly available and can be found at: http://software.broadinstitute.org/gsea/msigdb/search.jsp. Nominal *P* values were calculated based on 1000 random genes permutations.

### 2.21 ROC plotter analysis

Correlation between pathological complete response (pCR) or relapse‐free survival (RFS) at 5 years and *IFNB1* expression in CTX‐treated BC patients was done using ROC plotter [34]. Patients were selected for either taxane or anthracycline treatment and the relative expression levels of *IFNB1* (208173_at) investigated in patients predefined as responders (R) or nonresponders (NR). No additional filter was applied, and no outliers were excluded from the analysis.

### 2.22 Dataset analysis

Relative expression of the IFNS was assessed in laser capture microdissection (LCM) datasets – GSE145848, GSE83591, GSE35019 and GSE9014. The median expression of the IFNS genes was calculated for each sample. For GSE145848, matched samples of normal stroma (NS), tumour stroma (TS) and tumour epithelia (TE) were available. For all other datasets, nonmatched samples were used. Furthermore, IFNS expression was also investigated in GSE25055, GSE25065 and METABRIC. For all datasets, tumour samples were acquired prior to patients undergoing any treatment. All patients that underwent CTX treatment were selected for further analysis. Patients were then either divided according to the PAM50 classification (Luminal A, Luminal B, Normal, HER2, Basal‐like) or according the oestrogen receptor (ER) expression (positive or negative) and expression levels analysed.

### 2.23 Kaplan–Meier relapse‐free survival analysis

Survival analysis was done using compiled BC datasets of patients treated with neoadjuvant CTX (GSE25055 and GSE25065). Patients were treated with an anthracycline‐taxane CTX regimen. Patients from both datasets were pooled together and divided according the ER expression. The expression of the IFNS was assessed for each patient by calculating the median expression of all genes. Samples were then divided into IFNS high and low based on quartile cut‐off. Patients within the lower and upper quartile of expression were classified has IFNS^low^ and IFNS^high^, respectively. Furthermore, expression of the IFNS in patients with an event (relapse) within the first 2 years was compared to patients with no event (no relapse) in the same timeframe. This analysis was done for both ER‐positive and ER‐negative patients.

### 2.24 Statistics and reproducibility

Statistical analysis was performed using graphpad prism software v8 (Graphpad Software Inc, La Jolla, CA, USA). Graphs are shown as mean ± SEM, and each dot shown represents an independent biological replicate. Statistical analysis was performed in experiments with *n* ≥ 3. Statistical tests used in experiments are reported in figure legends. *P* values < 0.05 were considered statistically significant and statistical tests were two‐tailed. For Kaplan–Meier analyses of BC patients, statistical differences in survival curves were calculated by log‐rank (Mantel–Cox) test.

### 2.25 Ethics declaration (approval and consent to participate)

All human BC tissues used in this study were approved by the medical faculty of the University Hospital Tübingen (ethical vote: 150/2018BO2) and by the Medical Faculty of Heidelberg (ethical vote: S‐392/2015). Written informed consent was obtained from each patient prior to sample collection.

## 3 Results

### 3.1 Fibroblasts promote the recovery of cancer cells after high doses of chemotherapy

**Fig. 1:**
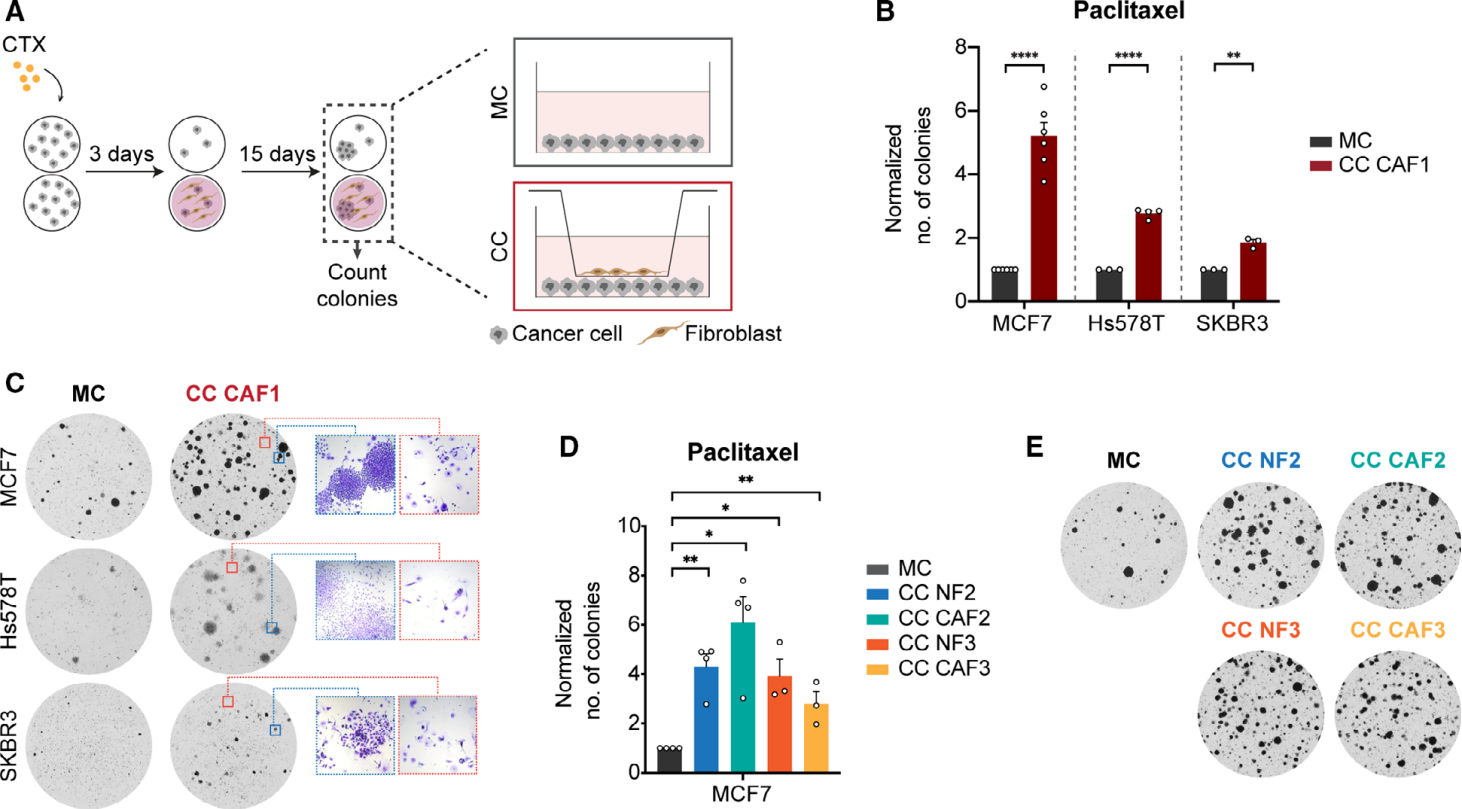
Fibroblasts promote the recovery of cancer cells after high‐dose CTX. (A) Schematic overview of recovery assay. Cancer cells were treated for 3 days with IC90 doses of CTX and allowed to recover for 2 weeks in the absence (MC) or presence (CC) of fibroblasts. Fibroblasts were seeded in trans‐wells and communicated with cancer cells via secreted factors as depicted in scheme. (B, C) Quantification of BC cell lines colonies in MC and CC with CAF1 at the end of recovery (15 days after drug withdrawal = day 3 + 15) after treatment with IC90 doses of paclitaxel (B). Each dot represents an independent biological replicate (MCF7: *n* = 6, Hs578T: *n* = 4, SKBR3 *n* = 3). Representative pictures of wells used for colony quantification are shown in C. Crystal violet staining under microscope (10× magnification) showing an area containing viable, proliferating colonies and arrested cells are shown in the blue and orange squares, respectively. (D, E) Recovery assay of MCF7 exposed to 4 nm paclitaxel in MC or CC with CAF2/NF2 and CAF3/NF3. Quantification of the number of colonies at the end point is shown in D and representative pictures of the wells in (E). Four independent biological replicates (*n* = 4) were done for fibroblast pair #2 and three independent replicates (*n* = 3) for fibroblast pair #3. Data are shown as mean ± SEM. *P* values were calculated using unpaired two‐tailed *t*‐test on biological replicates. *P < 0.05, **P < 0.01, ****P < 0.0001. Each dot represents an independent replicate.

Fibroblasts are important components of the TS, including in BC where they are one of the most prominent cell type surrounding neoplastic cells [35, 36] and have been shown to support tumour growth and metastasis [37, 38]. We sought to investigate whether stromal fibroblasts are capable of affecting the fate of cancer cells after exposure to chemotherapeutic agents. For this, we used primary fibroblasts isolated from BC patients from either tumour sites (CAF) or nonmalignant sites (NF). The fibroblast identity of the primary cells used was validated, and a representative image is shown in Fig. S1A. Additionally, the expression of the commonly used CAFs marker, αSMA was also investigated (Fig. S1B,C). Initially, the response curves of the BC cell lines MCF7, Hs578T and SKBR3 to commonly used CTX drugs – epirubicin and paclitaxel – were determined (Fig. S1D). Cancer cells exposed to high CTX doses were allowed to recover either in MC or indirect CC with primary fibroblasts isolated from BC samples as shown in Fig. 1A. Briefly, cancer cells were treated with IC90 doses of CTX for 3 days after which the drug was removed, and they were either cultured in the presence (CC) or absence (MC) of fibroblasts (recovery period). Treatment of MCF7, Hs578T and SKBR3 with CTX resulted in the long‐term recovery of a very small fraction of cells (below 0.1% of total seeded cells in the MC condition). Analysis of the number and size of the cancer cells colonies revealed that CC with all primary fibroblast lines tested significantly increased the number of cells that recovered after paclitaxel treatment (Fig. 1B–E, Fig. S1E). This was independent if fibroblasts had been isolated from a nonmalignant or a tumour site (NF or CAF, respectively). A similar effect in the recovery of cancer cells treated with epirubicin was observed (Fig. S1F–G). On the other hand, CC of untreated MCF7 with CAF1 did not increase the colony formation potential of cancer cells (Fig. S1H).

To determine whether the cells that recovered after CTX treatment were resistant to the treatment and whether CC with fibroblasts affected their response to the treatment, colonies from both MC (Col‐MC) and CC (Col‐CC) that survived paclitaxel treatment were expanded and re‐seeded for drug response assays (Fig. S2A). These assays revealed that colonies derived from MC and CC responded in a very similar fashion to paclitaxel treatment not only between each other but also when compared to the parental cell line (Fig. S2B,C), suggesting that recovery after treatment was not associated with resistance.

Combined these results indicate that the presence of stromal fibroblasts promotes the survival and re‐growth of nonresistant cancer cells after CTX treatment. To investigate the underlying mechanisms, additional phenotypic assays and RNA sequencing of both cancer cells and fibroblasts were performed.

### 3.2 Fibroblasts drive cell cycle re‐entry of cancer cells after chemotherapy

**Fig. 2:**
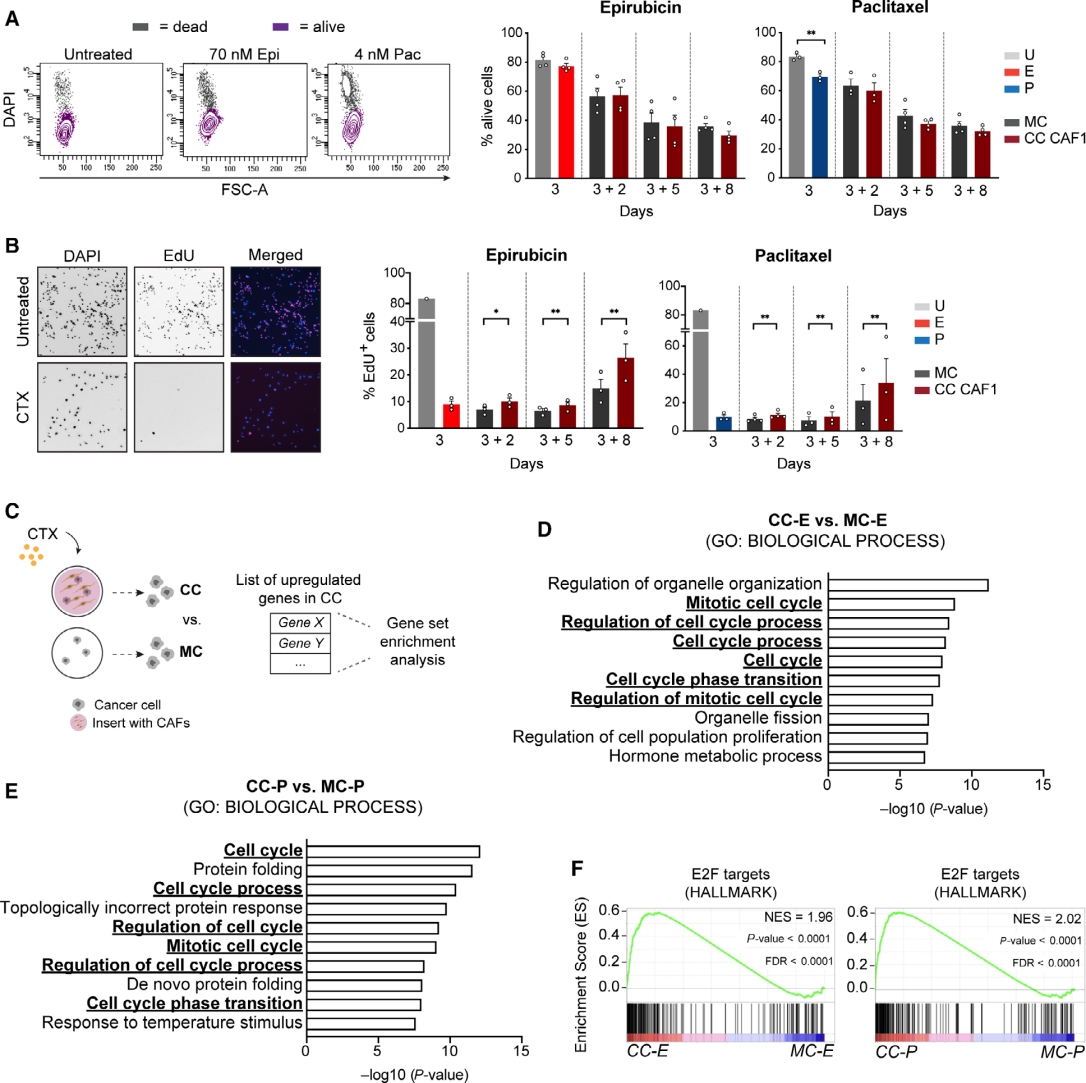
Fibroblasts promote the re‐entering in cell cycle of cancer cells after CTX treatment. (A) Flow cytometry analysis of apoptosis levels in cancer cells. DAPI‐negative cells were quantified using flow cytometry, as seen in the representative plot shown on the left panel. Apoptosis measurement of MCF7 in MC and CC with CAF1 (CC CAF1). Single cells negative for DAPI staining were counted and quantified by flow cytometry. Graphs show % of cells alive after treatment with either epirubicin (left) or paclitaxel (right) (day 3) and at different time points of recovery – after two (3 + 2), five (3 + 5) or eight (3 + 8) days of drug withdrawal. (B) Representative pictures of the EdU labelling assay showing DAPI and EdU staining in untreated and CTX‐treated MCF7. Graphs show % of MCF7 cells going through S‐phase after treatment with epirubicin (left) and paclitaxel (right; day 3) and at different time points of recovery. % of EdU‐positive cells (Edu^+^) was calculated by dividing labelled cells by total cells (DAPI staining). In A and B, data are shown as mean ± SEM, *P* values were calculated using ratio paired two‐tailed *t*‐test in biological replicates. *P < 0.05, **P < 0.01. Each dot represents an independent replicate (*n* ≥ 3). (C–E) Schematic overview of GO analysis (C). (D) GO analysis of upregulated genes in epirubicin‐treated MCF7 in CC with CAF1 (compared to MCF7 in MC) (*n* = 190 genes) for function (GO: Biological process) – top 10 list. E: GO (BP) analysis of upregulated genes in paclitaxel‐treated MCF7 in CC with CAF1 (compared to MCF7 in MC) (*n* = 140 genes) – top 10 list. Terms related to cell cycle are underlined and highlighted in bold. (F) Enrichment of proliferation signatures E2F targets – HALLMARK, in epirubicin (E)‐ and paclitaxel (P)‐treated MCF7 in CC with CAF1 compared to MCF7 in MC. *P* values were determined by random permutation tests.

Fibroblasts can modulate different aspects of tumour progression. Several studies have shown that the presence of fibroblasts can prevent cancer cells from undergoing programmed cell death after CTX treatment [5, 39]. To investigate whether CC with fibroblasts modulated the levels of apoptosis of cancer cells during the recovery period, the number of apoptotic cells was measured at different time points. DAPI labelling was used as a readout of cell death and the DAPI‐negative fraction was quantified by flow cytometry (Fig. 2A left panel). No difference in the percentage of alive cancer cells between MC and CC was observed in any of the cell lines tested (Fig. 2A right panel and Fig. S3A), indicating that fibroblasts are not protective against apoptosis. To further explore the role of fibroblasts during recovery, cell cycle profiling of cancer cells was done with EdU labelling (Fig. 2B left panel). This analysis revealed an increase in the percentage of cancer cells going through S‐phase in the presence of fibroblasts (Fig. 2B right panel and Fig. S3B). In addition, the number of senescent MCF7 cells was reduced in CC conditions during the recovery period (Fig. S3C). Finally, analysis of the different cell cycle phases using the FUCCI system [40] also supported these findings. Very briefly, cells with red nuclei [chromatin licensing and DNA replication factor 1 (Cdt1)‐RFP] were counted as G1, while cells with green nuclei (Geminin‐GFP) were counted as S‐G2‐M (Fig. S3D). After 8 days of CC with CAF1, there was a reduction in the percentage of cells expressing Cdt1 which was accompanied by an increase in the fraction of GFP‐positive nuclei, pointing to a decrease in the number of G1 arrested cells (Fig. S3E). These data indicate that fibroblasts promote the cell cycle re‐entry of cancer cells after treatment with high doses of CTX.

To further explore the crosstalk between cancer cells and fibroblasts in this context, we performed transcriptomic analysis by RNA sequencing of both MCF7 and CAF1 at an early time point of the recovery period (3 days after drug removal; see Fig. S4A for experimental layout). First, we analysed the transcriptome of cancer cells and explored how gene expression was affected by the treatment with cytotoxic agents. Next, the impact of CC in cancer cells was investigated. Treatment of MCF7 with epirubicin and paclitaxel led to the upregulation (fold change > 2, *P* < 0.05) of 2513 and 1533 genes, and downregulation (fold change < 0.5, *P* < 0.05) of 1014 and 338 genes, respectively (Fig. S4B). The vast majority of deregulated genes were shared by both treatments, pointing to the acquisition of a common transcriptional profile by cancer cells after exposure to the two cytotoxic agents. Several genes involved in DNA damage sensing pathways, including the p53 pathway, were upregulated in treated conditions compared to untreated while downregulated genes included genes involved in cell cycle progression (e.g. mitotic genes such as *BUB1* and *TTK*), confirming the induction of a proliferation arrest in cancer cells by both treatments (Fig. S4C). In order to investigate the impact of fibroblasts in CTX‐treated cancer cells, gene ontology (GO: *BIOLOGICAL PROCCESS*) term enrichment analysis of the transcriptome of CTX‐treated MCF7 in MC vs CC was done (Fig. 2C for schematic layout). Analysis of the upregulated genes (fold change > 1.2, *P* < 0.05) in epirubicin‐treated (*n* = 190) and in paclitaxel‐treated cancer cells (*n* = 140) in CC with CAF1 compared to their MC counterparts revealed that the presence of fibroblasts (CC) induced a signature of genes involved in cell cycle progression in cancer cells (Fig. 2D,E for epirubicin and paclitaxel treatment, respectively), confirming the results from the phenotypic assays (Fig. 2B). This signature was not observed in untreated cancer cells that had been in CC with fibroblasts (Fig. S4D). In addition, GSEA (HALLMARK collection) showed that in CTX‐treated MCF7 in CC with CAF1, but not untreated MCF7, there was an enrichment in proliferation signatures (Fig. 2F; See Table S5 for list of top 10 HALLMARKS in three conditions (epirubicin – E, paclitaxel – P, untreated – U) with false discovery rate [(FDR) < 0.05]. Indeed, in the epirubicin and paclitaxel conditions, E2F targets signature was the first and second enriched gene set, respectively.

The above findings suggest a model in which stromal fibroblasts promote the cell cycle re‐entry of cancer cells, likely by providing them with factors that allow cancer cell survival and proliferation after treatment with chemotherapeutic agents.

### 3.3 Chemotherapy‐treated cancer cells induce an anti‐viral state in fibroblasts

**Fig. 3:**
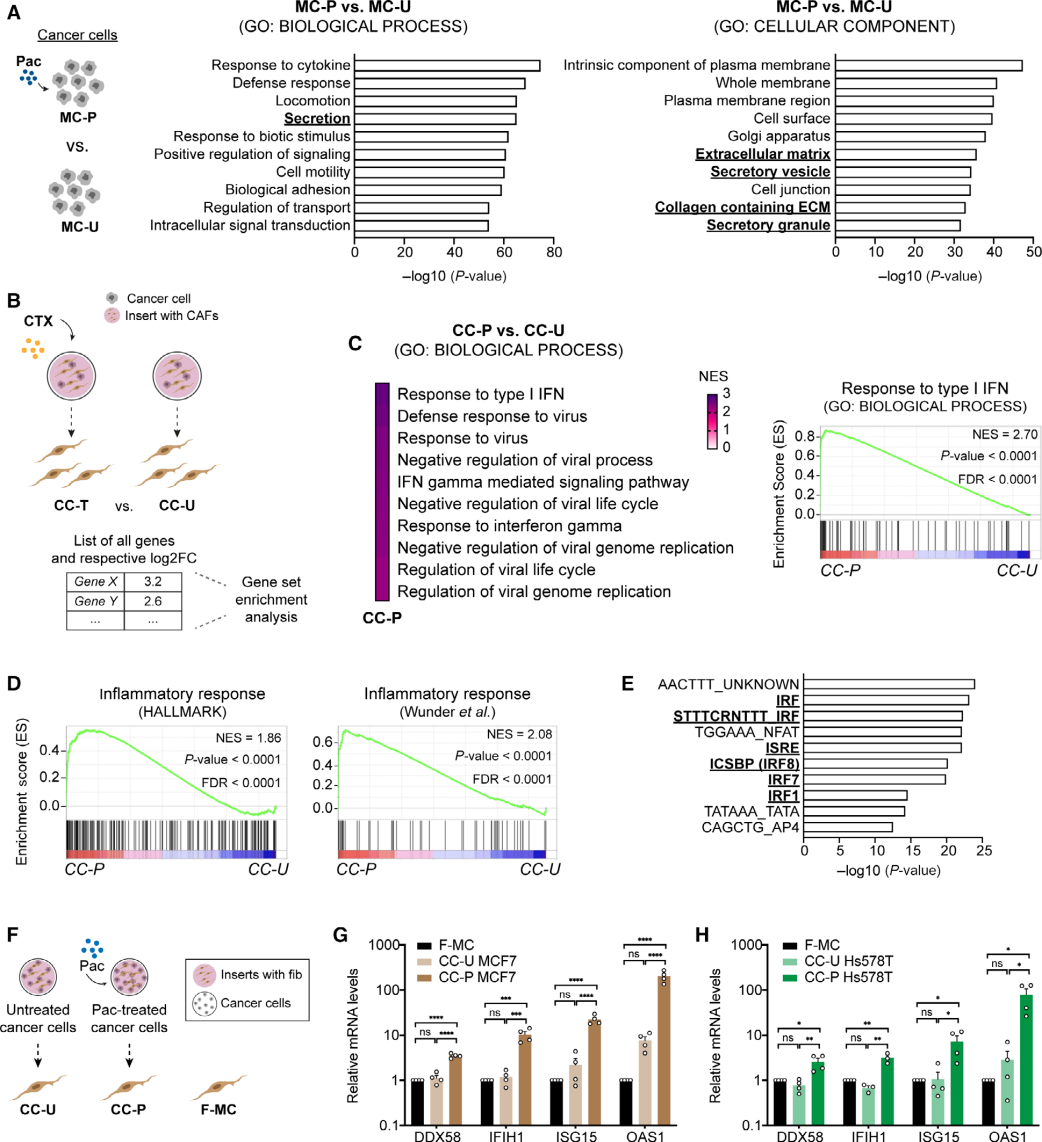
Fibroblasts acquire an anti‐viral state after CC with CTX‐treated cancer cells. (A) Top 10 enriched GO terms for biological process (BP) (left panel) and cellular component (CC) (right panel) of top 2000 upregulated genes in paclitaxel‐treated MCF7 (MC‐P) compared to untreated (MC‐U). Terms related to secreted factors are highlighted in bold. (B, C) Schematic overview of enrichment analysis. Comparison between fibroblasts in CC with CTX‐treated (CC‐T) and untreated (CC‐U) cancer cells was done (B). (C) GSEA analysis of CAF1 in CC‐P compared to CC‐U. Top 10 significantly enriched GO terms in CC‐P enriched are shown. Enrichment curve for the top biological process (Response to type I interferon) is shown in right panel. *P* values were calculated by random permutation tests. (D) CAF1 in CC with paclitaxel‐treated cancer cells (CC‐P) acquire an inflammatory signature. Enrichment analysis revealed in the top 10 hallmark signatures (HALLMARK), the inflammatory response gene set (left panel). An independent gene set for inflammation [41] was used for GSEA (right panel). (E) Top 10 over‐represented transcription factors and motifs involved in the regulation of genes upregulated (*n* = 775 genes) in CAF1 in CC with paclitaxel‐treated cancer cells compared to CAF1 in CC with untreated cancer cell. Transcription factors known to be involved in antiviral response are underlined and highlighted in bold. (F) Schematic overview of experimental conditions. CAF1 was grown in MC (F‐MC), in CC with paclitaxel‐treated cancer cells (CC‐P) or in CC with untreated cancer cells (CC‐U). (G, H) Validation of antiviral like state in CAF1 in CC with MCF7 (G) and HS578T (H) (*n* = 4 for each cell line). Expression of *DDX58*, *IFIH1*, *ISG15* and *OAS1* in CAF1 was investigated by qRT‐PCR and was normalized to F‐MC. CAF1 was co‐cultured for 5 days with cancer cells. Data are shown as mean ± SEM, *P* values were calculated using one‐way ANOVA in biological conditions. *P < 0.05, **P < 0.01, ***P < 0.001, ****P < 0.0001. Each dot represents an independent replicate.

Comparison between the transcriptomes of untreated vs treated cancer cells showed that exposure of cancer cells to CTX induced profound changes in the expression of numerous genes, as described in the previous section. GO analysis for both function (GO: BIOLOGICAL PROCESS) and localization (GO: CELLULAR COMPONENT) of the top 2000 upregulated genes in paclitaxel‐ and epirubicin‐treated MCF7 revealed that these gene sets were enriched for secreted factors (Fig. 3A and Fig. S5A for paclitaxel and epirubicin‐treatment, respectively), pointing to the reprogramming of the secretory profile of cancer cells after CTX treatment [7]. To understand how the CTX‐induced secretome of cancer cells affected the fibroblasts that were expose to it, we next focused our analysis on the transcriptome of fibroblasts and investigated gene expression changes when fibroblasts were in CC with untreated vs treated cancer cells (Fig. 3B for schematic layout). Comparison between these conditions revealed a strong induction of an antiviral response in fibroblasts in CC with cancer cells treated with either paclitaxel or epirubicin (Fig. 3C and Fig. S5B, respectively). This antiviral state in fibroblasts was accompanied by the acquisition of an inflammatory response signature, which was among the top 10 enriched signatures using the HALLMARK gene sets (Fig. 3D and Fig. S5C–D). Furthermore, this inflammatory state was also validated using an independent gene set [41] (Fig. 3D and Fig. S5C,D). We also investigated which transcription factors and motifs might be involved in the regulation of the upregulated genes (fold change > 1.5, *P* < 0.05) in the fibroblasts in CC with CTX‐treated cancer cells (775 and 295 genes in paclitaxel and epirubicin, respectively) using the transcription factor targets (TFTs) collection for GSEA. This identified interferon regulatory factors (IRF) and interferon responsive sequence element motif as the top hits (Fig. 3E and Fig. S5E). These findings indicate that CTX‐treated cancer cells trigger the acquisition of an anti‐viral state in fibroblasts characterized by the expression of numerous inflammatory modulators including several ISGs.

To validate these results, we used reverse transcription with quantitative PCR (RT‐qPCR) to investigate the expression of a subset of genes (*DDX58, IFIH1, ISG15* and *OAS1*) that were discovered significantly upregulated in both treatments (Fig. S5F) and that have been described to be involved in viral responses [42] (See Fig. 3F for schematic layout). In addition to their established role in viral response, these genes were selected based on the number of reads in the RNA‐sequencing data, since the detection of low expressed genes using RT‐qPCR can be challenging. Strikingly, the expression of these genes in CAF1 was not significantly induced when fibroblasts were in CC with untreated cancer cells (Fig. 3G,H). In contrast, treatment of cancer cells with paclitaxel prior to the CC led to a strong increase in the expression of all four anti‐viral genes (Fig. 3G,H), validating the results from RNA sequencing. It has been previously demonstrated that treatment of primary fibroblasts with high doses of CTX can induce a pro‐inflammatory state in fibroblasts [43]. Notably, in this context CTX treatment of CAF1 did not induce the expression of any of the ISGs analysed, except for *OAS1* upon paclitaxel treatment (Fig. S5G).

Taken together, we show that fibroblasts are reprogrammed by CTX‐treated cancer cells. Fibroblasts in this context acquire a similar transcriptional profile to cells exposed to viruses with increased expression of several pro‐inflammatory modulators.

### 3.4 Antiviral state in fibroblasts is independent of nucleic acid sensing pathways

We next sought to determine the mechanism that drives the antiviral state in fibroblasts. To understand if the transcriptional reprogramming of fibroblasts was induced directly by factors secreted by cancer cells after treatment with CTX or if the crosstalk between cancer cells and fibroblasts played any role in promoting the expression of antiviral genes, the supernatants from untreated or paclitaxel‐treated cancer cells were collected and directly added to fibroblasts (Fig. S6A). As in all previous experiments, the chemotherapeutic drugs had been removed after 3 days and were not present in the media at the time the supernatants (72 h after drug removal) were added to the fibroblasts. The exposure of CAF1 to the supernatant of both MCF7 and Hs578T previously treated with IC90 doses of paclitaxel was sufficient to induce the expression of the antiviral genes (Fig. S6B). As before, this increase was absent in fibroblasts exposed to the CM of untreated cancer cells. Furthermore, upregulation of the antiviral genes was observed in fibroblasts that had been isolated from both nonmalignant and tumour tissue, indicating that these were equally reprogrammed by the secretome of paclitaxel‐treated cancer cells (Fig. S6C). This analysis supports the concept that chemotherapeutic drugs modify the secretome of cancer cells in BC. We further demonstrate that this newly acquired TCS can then affect their communication with the microenvironment and highjack the fibroblasts that are exposed to it to induce the antiviral state.

**Fig. 4:**
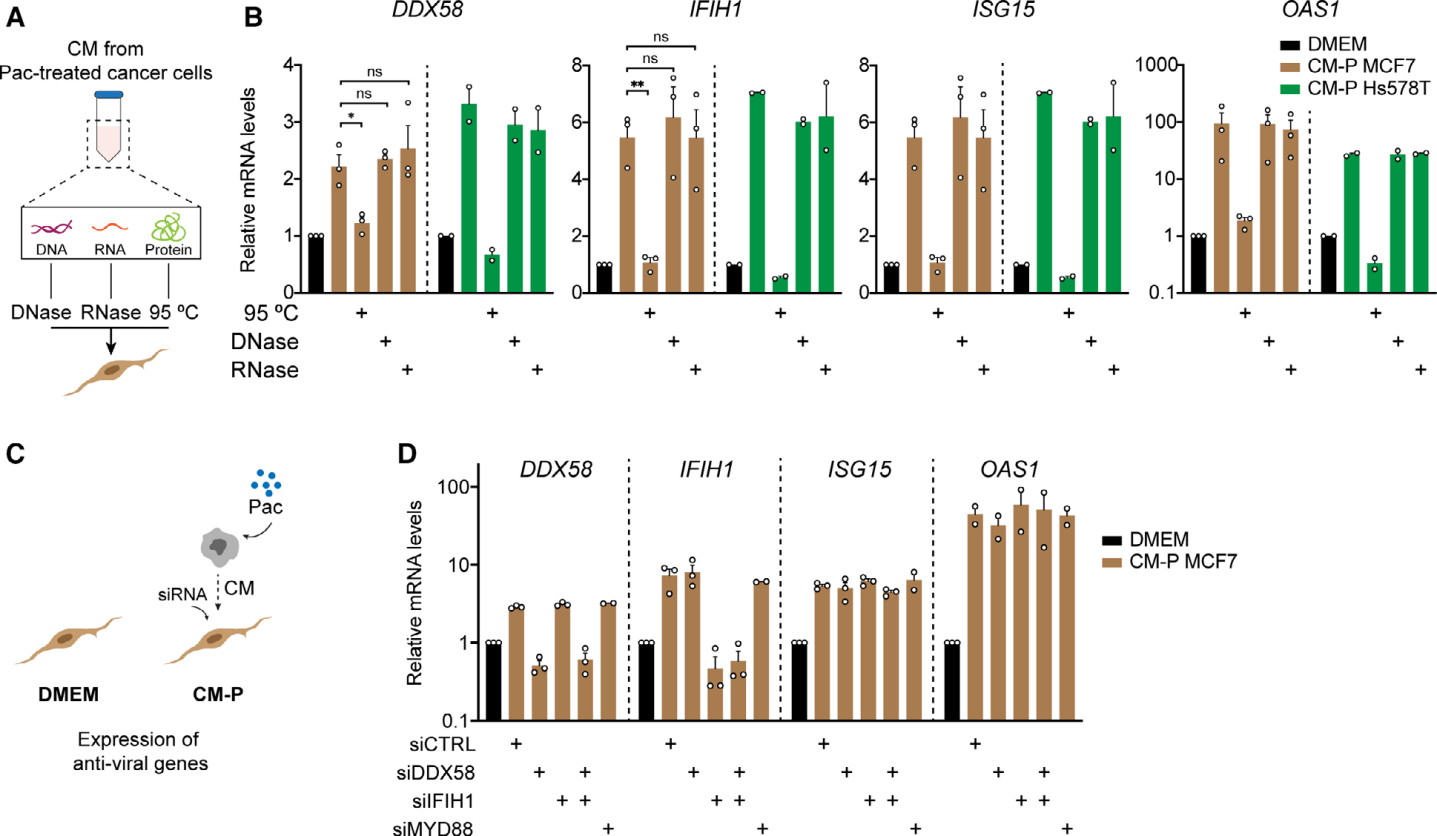
Antiviral like state in fibroblasts is independent of nucleic acid sensing pathways. (A) Schematics of the different treatments to which the supernatant of cancer cells was exposed. Collected CM (72 h CM) from treated cancer cells was either exposed to DNase, RNase or 95 ºC treatment before addition to fibroblasts. Fibroblasts were exposed to the CM for 48 h. (B) RT‐qPCR analysis of anti‐viral genes in CAF1 after exposure to the CM without any treatment, after boiling (95 ºC), after DNASE or RNASE treatment. CM from both MCF7 (shown in brown bars; *n* = 3) and Hs578T (shown in green bars; *n* = 2) treated with 4 and 8 nm paclitaxel, respectively, was used. (C) Layout of experimental setup for nucleic acid sensing receptors KD experiments. (D) KD of nucleic acid sensing pathways in CAF1 and its impact in the expression of anti‐viral genes measured by RT‐qPCR. Each dot represents an independent biological replicate. Data is shown as mean ± SEM, *P* values for experiments with *n* > 2 were calculated using one‐way ANOVA in biological replicates. *P < 0.05, ** *P* < 0.01 ns not significant.

Previous studies show that the expression of antiviral genes can result from the activation of multiple pathways. The two main nucleic acid sensing pathways are activated by RIG‐I and Toll‐like receptors, which directly bind and sense viral DNA or RNA. In the tumour context these pathways can be activated by exosomes [5] or by DAMPs derived from cancer cells which can then be recognized by neighbouring cells [44]. To understand whether these pathways were involved in the induction of the antiviral like state in the fibroblasts, the supernatants from MCF7 and Hs578T treated with paclitaxel were collected and subjected to different treatments (See Fig. 4A for schematic layout). RNase and DNase treatment of the CM did not have any impact in the expression of antiviral genes in the fibroblasts. In contrast, incubation of the CM at 95 ºC for five minutes completely abolished the upregulation of the four antiviral genes (Fig. 4B). Additionally, the supernatant collected from paclitaxel‐treated cancer cells was submitted to ultracentrifugation and the exosome‐enriched (Exo^+^) and exosome‐depleted (Exo^−^) fractions were collected and added to fibroblasts. Only the exosome‐depleted fraction was capable of inducing the expression of *DDX58*, *IFIH1*, *ISG15* and *OAS1* to a similar extent as the ‘full’ CM (Fig. S6D). These results suggest that the antiviral state is not induced by exosomes or DAMPs secreted by cancer cells after CTX treatment but rather by a soluble protein factor. To further confirm these observations, we took the complementary approach of silencing the nucleic acid sensing pathways in fibroblasts using RNA‐interference (See Fig. 4C for schematic layout). Knockdown (KD) of one or both genes encoding the RNA cytosolic receptors of the RIG‐I pathway – *DDX58* and *IFIH1* – had no impact in the anti‐viral like state (Fig. 4D and Fig. S6E for KD efficiency). Furthermore, silencing of *MYD88*, an important mediator of Toll‐like receptor signalling, was also not enough to abolish the induction of the anti‐viral genes in fibroblasts (Fig. 4D and Fig. S6E for siRNA silencing efficiency).

Collectively, these data indicate that fibroblasts are modulated by a soluble protein factor that is secreted by cancer cells after CTX treatment rather than by nucleic acids or exosomes that activate nucleic acid sensing pathways.

### 3.5 Chemotherapy‐induced secretion of IFNβ1 by cancer cells induces antiviral state in fibroblasts and promotes cancer cell recovery

**Fig. 5:**
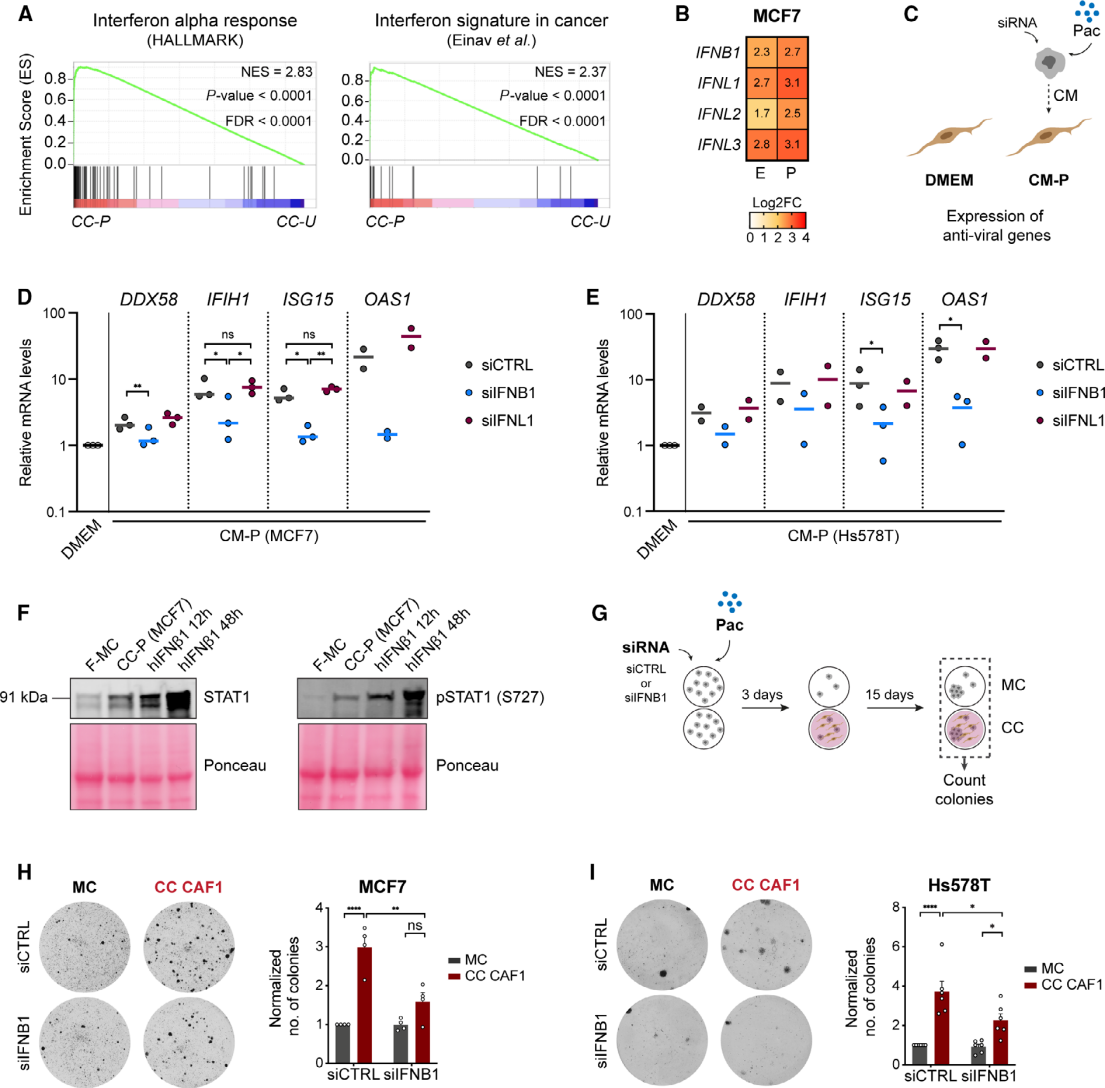
IFNβ1 secreted by CTX‐treated cancer cells drives fibroblasts into an antiviral state and promotes recovery. (A) GSEA for HALLMARK showed interferon response as number one enriched term (left panel) in fibroblasts in CC with paclitaxel‐treated cancer cells (CC‐P) compared with untreated cancer cells (CC‐U). Enrichment analysis using an independent gene set for IFNS in cancer [45] was also done (right panel). *P* values were determined by random permutation tests. (B) Heat‐map with expression of significantly upregulated interferon transcripts in MCF7. Results for epirubicin (E)‐ and paclitaxel (P)‐treated cancer cells are shown in log2 fold change and are normalized to untreated MCF7. (C) Layout of experimental setup for interferon KD experiments. (D, E) *DDX58*, *IFIH1*, *ISG15* and *OAS1* expression in CAF1 determined by RT‐qPCR. CM from paclitaxel‐treated MCF7 (*n* = 3, except for *OAS1* where *n* = 2) (D) and HS578T (*n* = 2, except for *ISG15* and *OAS1* where *n* = 3) (E) transfected with a control siRNA (siCTRL), a siRNA against *IFNB1* (siIFNB1) or a siRNA against *IFNL1* (siIFNL1) was collected and added to CAF1. Relative fold change is normalized to CAF1 grown in DMEM. mRNA levels were normalized against two house‐keeping genes (*ACTB* and *PUM1*). Each dot represents an independent experiment. *P* values for *n* > 2 were calculated using one‐way ANOVA in biological replicates. *P < 0.05, **P < 0.01, ***P < 0.001. (F) Immunoblot of STAT1 and pSTAT1 (S727) in CAF1 lysates in MC (F‐MC), in CC with paclitaxel‐treated MCF7 (CC‐P) for 5 days or stimulated with 100 ng·mL^−1^ of recombinant human IFNβ1 (hIFNβ1) for 12 or 48 h. Ponceau was used as loading control. (G–I) Schematic overview of recovery assay after gene KD (G). (H, I) Colonies in recovery assay of MCF7 (H) and HS578T (I) transfected with a siCTRL or a siRNA against *IFNB1* (siIFNB1). All conditions were normalized to siCTRL MC. Representative pictures of wells at the end point and their quantification are shown on the left and right panels, respectively. Each dot represents an independent replicate (*n* = 4 for MCF7 and *n* = 6 for HS578T). *P* value was calculated using one‐way ANOVA in biological replicates. *P < 0.05, **P < 0.01, ****P < 0.0001. Data are shown as mean ± SEM.

Based on the finding that the antiviral state in fibroblasts was mediated by a protein and not by DNA‐ or RNA‐sensing pathways, we continued to explore our transcriptomic data to identify candidate factors. A strong enrichment in interferon (IFN) response signalling in fibroblasts in CC with CTX‐treated cancer cells was observed (Fig. 3C and Fig. S5B; Fig. 5A and Fig. S7A for paclitaxel and epirubicin, respectively. For a list of top 10 HALLMARK terms, see Table S6). IFN response was the top enriched gene set identified in the HALLMARK analysis, and further validation was done using an independent gene set [45]. Comparison between the expression of IFN transcripts in untreated MCF7 cells and their treated counterparts showed a strong upregulation of type I – *IFNB1* – and type III – interferon lambdas 1, 2 and 3 (*IFNL1*, *IFNL2*, *IFNL3*) – IFNs after treatment (Fig. 5B). No reads were detected for the majority of other IFNs, including all 13 isoforms of interferon alpha (*IFNA*) and *IFNG*. Upregulation of *IFNB1* and *IFNL1* was validated by RT‐qPCR in MCF7 and HsS578T (Fig. S7B). Both type I and type III IFNs are potent inducers of the antiviral state and are known to trigger the expression of ISGs. However, contrary to type I IFN, which is known to mediate this state in any type of tissue due to the ubiquitous expression of its receptor – the IFNAR1‐IFNAR2 heterodimer – type III IFN activity seems to be compartmentalized to epithelial cells [46]. Type III interferon receptor is formed by the heterodimer of IL10RB and IFNLR1. The latter receptor is reported to only be expressed by epithelial cells [46]. Accordingly, no reads were detected in fibroblasts in our RNA sequencing (data not shown). Nonetheless, we modulated the expression of both IFNβ1 and IFNλ1 in cancer cells in order to understand whether they were responsible for the activation state observed in fibroblasts (Fig. 5C). Blockade of IFNβ1 by either RNA‐interference or a blocking antibody led to an almost complete abrogation of the upregulation of anti‐viral genes in fibroblasts (Fig. 5D,E and Fig. S7C–E for siRNA silencing efficiency), whereas silencing of IFNλ1 in cancer cells had no effect (Fig. 5D,E, Fig. S7D,E for siRNA silencing efficiency). After binding to its receptor, type I IFN induces a cascade of events via JAK proteins that culminates in the phosphorylation of the transcription factor STAT1. Activated STAT1 then translocates to the nucleus where it forms a complex with STAT2 and IRF9 named ISGF3, which induces the expression of IFN target genes, including *STAT1* itself [8]. There was indeed a strong upregulation in the expression of STAT1 as well as its activation marker – serine 727 phosphorylation (STAT1‐S727) [47] – in CAF1 in CC with paclitaxel‐treated MCF7 (Fig. 5F). Consistent with these results, depletion of one of the IFNβ1 receptor subunits – IFNAR1 – in fibroblasts, similarly abrogated the anti‐viral state as well as the increase in expression of STAT1 in CAF1 in CC‐P (Fig. S7F–G,J for siRNA silencing efficiency). This was further confirmed with two independent siRNAs targeting siIFNAR1 (Fig. S7H,J for KD efficiency). We next examined the influence of STAT1 and STAT2, two transcription factors reported to be involved in type I IFN signalling, on the expression of the anti‐viral genes. KD of STAT1 by siRNA had no effect on the expression of the antiviral genes (Fig. S7I left panel and Fig. S7J), indicating that the observed effect was independent of STAT1. Since STAT2 can form a complex with IRF9 and induce the expression of ISGs in the absence of STAT1, which seems to be tissue‐ and context‐dependent [48], we next silenced STAT2 in CAF1 before adding the CM from treated cancer cells. This KD of STAT2 prevented the induction of the antiviral genes in the fibroblasts (Fig. S7I right panel and Fig. S7J), indicating that here STAT2 might play an important role in the regulation of the antiviral genes.

To test the impact of IFN in the recovery of cancer cells after CTX treatment, we then silenced IFNβ1 in cancer cells and investigated its impact on the colony formation potential of these cells after paclitaxel treatment (Fig. 5G). Depletion of IFNβ1 was sufficient to decrease the recovery capacity of cancer cells in the presence of fibroblasts (Fig. 5H,I), indicating that this paracrine communication between cancer cells and stromal fibroblasts plays an important role in promoting the re‐growth and survival of cancer cells after CTX treatment.

Together, we show that chemotherapeutic drugs induced the expression of IFNβ1 in cancer cells and that secreted IFNβ1 induced the expression of ISGs in fibroblasts. Importantly, this crosstalk promoted the recovery of cancer cells after CTX treatment. This indicates that cancer cell‐secreted IFNβ1 induces a pro‐tumorigenic state in fibroblasts that is associated with the expression of anti‐viral genes.

### 3.6 Clinical significance of IFNβ1 and anti‐viral state

**Fig. 6:**
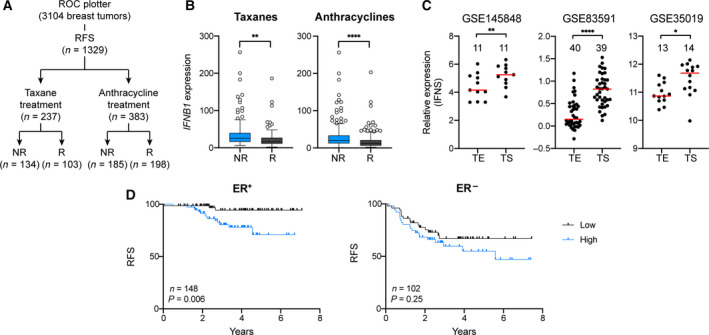
Clinical significance of IFNβ1 axis. (A) Schematic overview of patient number for each group in ROC plotter. (B) Analysis of *IFNB1* expression and RFS outcome using ROC plotter in BC patients treated with taxanes (B) or anthracyclines (C). *P* value was calculated using unpaired two‐tailed *t*‐test. **P < 0.01, ****P < 0.0001. NR, nonresponder, R, responder. Response is determined based on the first 5 years. Patients with an event (recurrence) within the first years are classified as NR, while patients with no event (no recurrence) are called responders. (C) Analysis in LCM datasets – GSE145848, GSE8391 and GSE35019 – of the IFNS expression in tumour epithelium (TE) and TS. Each dot represents one sample. Patient matched data were available in GSE145848. *P* values were then calculated using two‐tailed paired *t*‐test. **P < 0.01. For the other two datasets, no matched data was available and *P* values were calculated using two‐tailed unpaired *t*‐test. *P < 0.05, ****P < 0.0001. Red line represents median expression in each group. Numbers show total samples analysed in each group. (D) Kaplan–Meier analyses of BC patients, associating IFN signature (IFNS) with RFS. Patients were divided into ER‐positive (ER^+^) and ‐negative (ER^−^) and IFNS expression was calculated for each patient. Compiled data set from GSE25055 (ER^+^ *n*= 152; ER^−^ *n* = 115) and GSE25065 (ER^+^ *n*=106; ER^−^ *n* = 68). Lower and upper quartile cut‐off was used to group samples into low and high. *P* values were determined by log‐rank (Mantel‐Cox) test.

To explore the clinical significance of this newly discovered axis of communication between cancer cells and fibroblasts, publicly available datasets of patient data were analysed. To this end, we first investigated the impact of IFNβ1 expression in the outcome of BC patients treated with CTX, using ROC plotter [34]. This tool comprises the transcriptome data of a total of 3014 BC patients and their matching clinical information regarding RFS at 5 years (*n* = 1329) (Fig. 6A) and/or pCR (*n* = 1775; Fig. S8A). NR for RFS and pCR were defined by the authors as patients who relapsed within 5 years or had residual tumour tissue after treatment, respectively. We looked at the correlation between *IFNB1* expression and RFS or pCR. Patients from all subtypes were included in the analysis. Achievement of pCR in patients treated with taxanes (*n* = 1213) or anthracyclines (*n* = 1626) did not correlate with the levels of *IFNB1* (Fig. S8B). In contrast, higher levels of *IFNB1* were associated with a worse outcome in RFS in patients undergoing treatment with both taxanes (*n* = 237) and anthracyclines (*n* = 383; Fig. 6B and Fig. S8C), indicating an inverse correlation between RFS time and *IFNB1* expression.

Fibroblasts in CC with CTX‐treated cancer cells had 250 commonly upregulated genes compared to their counterparts that were in CC with untreated cancer cells. According to GO analysis, 52 out of these 250 genes are involved in viral response (see Table S7 for list of genes). We thus wanted to explore the impact of this anti‐viral signature, which we named IFN signature (IFNS) in the outcome of BC patients undergoing CTX treatment. Since publicly available datasets containing clinical data mostly represent the transcriptomes of different cell types, comprising both tumour and stromal cells, we investigated the expression of this signature in LCM datasets to understand the contribution from cancer and stromal cells to the expression of these genes. In datasets containing tumour epithelium (TE) and TS samples, the expression of the IFNS was significantly higher in the stromal compartment than in cancer cells (Fig. 6C). This finding pointed to a stronger contribution of the stromal cells to the expression of these genes in whole tissue samples. Furthermore, using the LCM datasets we could also verify that this signature is upregulated in stroma in close proximity to the tumour site (TS) compared to stroma isolated from more distant areas (NS) counterparts (Fig. S8D), which could hint to the association of this signature with a pro‐malignant state of fibroblasts. Using publicly available data (GSE25055–GSE25065 [49] and METABRIC [50]) from BC patients that underwent CTX treatment, we observed that patients with more aggressive subtypes (Basal‐like) had higher expression of the IFNS (Fig. S8E). Furthermore, when patients were separated into ER‐positive (ER^+^) and ‐negative (ER^−^) subgroups, a higher expression of the signature in ER‐negative patients was observed (Fig. S8F). Next, BC patients were clustered in quartiles according to the expression of the antiviral signature identified in the fibroblasts. Since METABRIC lacks data on recurrence, we focused our analysis in the GSE25055–GSE25065 datasets, in which patients were treated in a neoadjuvant setting with an anthracycline taxane‐based regimen of CTX and RFS time information is available. Patients from GSE25055 and GSE25065 were pooled together, separated into ER^+^ and ER^−^ and then divided into IFNS^low^ and IFNS^high^, based on the lower and upper quartile, respectively. A clear inverse correlation between the expression of the IFNS and RFS time was observed in ER^+^ patients, whilst there was no significant impact in the ER^−^ ones (Fig. 6D). Finally, ER^+^ patients with recurrences within the first 2 years (NR) had significantly higher levels of the IFNS, further confirming the predictive value of the gene signature (Fig. S8G). While only 1% and 4% of the ER‐positive patients with low expression (lower quartile) of the signature relapsed within the first 2 years (one out of 74 ER‐positive patients) and 5 years (three out of 74), respectively, this value was around 8% (six out of 74) and 15% (11 out of 74) for the high IFNS expression (upper quartile) patients. No significant difference was seen in ER^−^ patients (Fig. S8G). IFNS^low^ (10 out of 51 ER‐negative patients) and IFN^high^ (16 out of 51) patients had around 19% and 31% recurrence rates within the first 2 years, respectively. The same trend was observed when analysing recurrences in the first 5 years in ER‐negative patients, with 29% of IFN^low^ having an event, compared to 39% of IFN^high^.

**Fig. 7:**
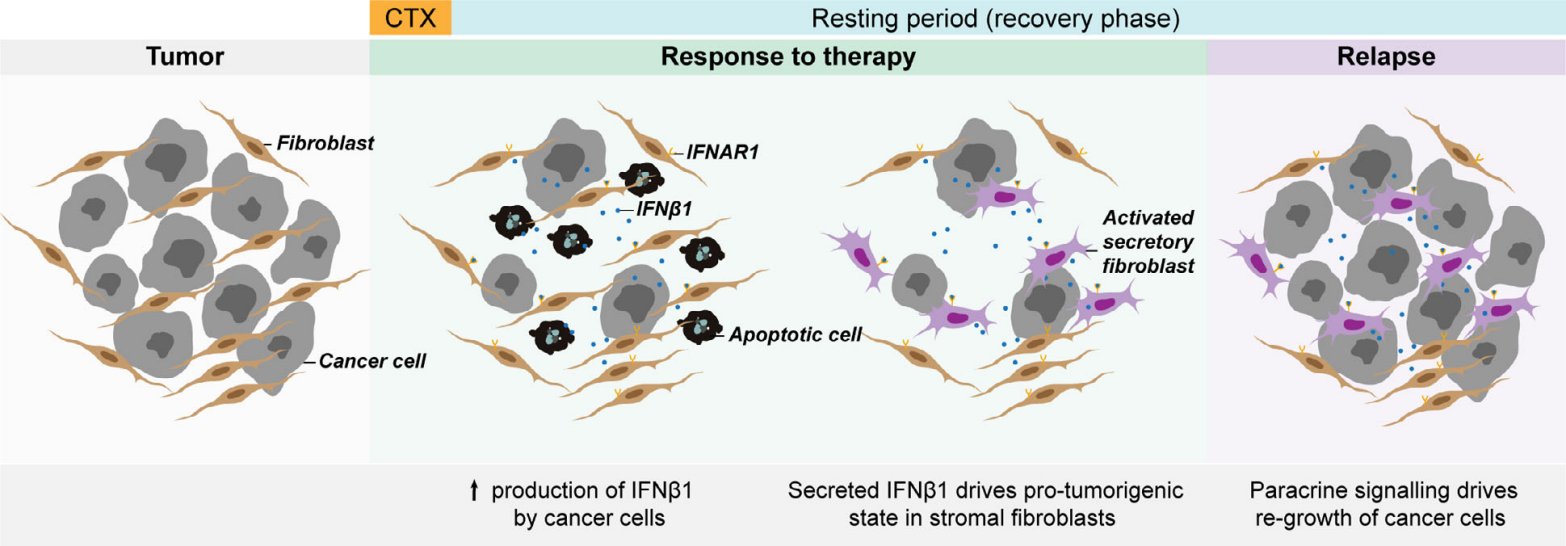
Schematic overview. Treatment with high doses of chemotherapeutic agents (CTX) induces the expression of IFNβ1 by cancer cells and its subsequent secretion into the tumour microenvironment. Stromal fibroblasts binding to IFNβ1 through IFNAR1 get activated and acquire an anti‐viral, pro‐inflammatory signature associated with the expression of several ISGs. Through this process, stromal fibroblasts are reprogrammed to a pro‐tumorigenic state leading to cancer cell recovery after CTX treatment and cancer relapse in patients with BC.

We show that cancer cells exposed to CTX upregulate the expression of IFNβ1 which is then secreted into the microenvironment and modulates the activation of fibroblasts. Fibroblasts exposed to IFNβ1 acquire an anti‐viral state that is characterized by increased expression of numerous inflammatory modulators, which can then promote the recovery of cancer cells after CTX (see Fig. 7 for schematic overview).

## 4 Discussion

Chemotherapy is still the standard of care for a large number of patients with cancer. In BC, a regimen based on anthracyclines and taxanes is commonly used [51]. Treatments are often given in cycles, in which patients are administered a maximum tolerable dose of the respective chemotherapeutic agent. The frequent failure of cancer cells to respond to therapy leads to tumour progression and ultimately results in the development of advanced metastatic disease, which is the main cause of death in cancer patients. An understanding of the mechanisms that drive therapeutic failure and development of resistance is of utmost importance and could help devising strategies to prevent relapses and metastatic disease. Cancer cells can evade therapy through a variety of mechanisms and the tumour microenvironment is known to play an important role in this process. In this study, we have investigated the impact of high chemotherapeutic doses on cancer cells, its influence in the crosstalk with stromal fibroblasts and how this communication affects the outcome of cancer cells to CTX treatment. Indeed, CAFs have been shown to drive the development of resistance of cancer cells to several therapies, including CTX [5, 6]. Here, we show that stromal fibroblasts are not only affecting the response of tumour cells to CTX by promoting the development of resistance, but they can also influence the fate of cancer cells after the exposure to cytotoxic agents. We provide evidence that in the presence of fibroblasts, cancer cells can recover from the cytotoxic damage induced by CTX and re‐start proliferating. This recovery potential is absent when cancer cells are grown alone. Additionally, we show that the cancer cells that recover in the presence of fibroblasts are not resistant to the agents that they were initially treated with. While the presence of resistant cells is one of the main drivers of therapeutic failure, it has been previously reported that persister cancer cells can escape the cytotoxic or cytostatic effect of treatment [3, 52]. Based on the results from our study, we hypothesize that fibroblasts help these cancer cells survive and re‐enter cell cycle, thereby facilitating the occurrence of relapses.

In recent years, the secretome of cancer cells and its impact on therapy response has gained attention and numerous studies explore its role in several aspects of tumour progression. Molecules released by cancer cells can strongly modulate not only other cancer cells but also their microenvironment [7]. Several factors can influence and alter the spectrum of secreted molecules by cancer cells, including a large number of conventional cancer therapies. For example, it has been described that CTX treatment can lead to the expression and release of several pro‐tumourigenic molecules, including interleukins such as interleukin 6 (IL‐6), whose expression negatively correlates with patient outcome [53, 54]. The expression of other molecules, such as type I IFN, has also been shown to be modulated by anti‐tumour therapies, especially radiation. Type I IFN signalling can have both anti‐tumourigenic and pro‐tumourigenic roles. Several studies demonstrate the importance of type I IFN signalling in effective anti‐tumour immune responses [20, 21, 55]. Nevertheless, the role of type I IFN in the context of cancer is still not clear. While strong evidence exists that type I IFN is essential in several tumour entities for effective therapy response and immune engagement against cancer cells, other studies seem to challenge this black and white view on this signalling pathway. Chen *et al*. [12] showed that depletion of type I IFN receptor, *IFNAR1*, in cancer cells promoted a strong immune response after treatment with radiation, indicating that the outcome of type I IFN signalling in tumour progression might be dependent on several factors, including in which compartment of the tumour this pathway is activated. Moreover, high expression of interferon‐regulated genes in several tumour entities also strongly correlates with a worse outcome in patients [56, 57]. All of these studies highlight the high degree of plasticity of type I IFN and the need for a better understanding of the role of this pathway in the context of cancer. While most of these studies focus on the impact of type I IFN in immune cells, much fewer reports addressing its effect in stromal fibroblasts are available. Of note, Hosein *et al*. [56] identified in BC a subset of fibroblasts characterized by a type I IFN response and showed that high expression of certain ISGs in patient samples correlated with patient outcome. In our study, we show that cancer cells exposed to chemotherapeutic agents upregulate the expression of type I IFN, namely IFNβ1. This effect was observed when either a taxane (e.g. paclitaxel) or an anthracycline (e.g. epirubicin) were applied. Additionally, exposure of stromal primary fibroblasts to the CM of CTX‐treated cancer cells triggered the acquisition of an antiviral, pro‐inflammatory state in fibroblasts that was characterized by the expression of multiple ISGs. The expression of ISGs in the context of cancer has been previously shown to be induced by nucleic acid sensing pathways [5, 22, 58]. However, here we do not find a direct link between these pathways and the inflammatory response observed in fibroblasts, but instead we show that this state is mediated by cancer cell‐secreted IFNβ1. Nonetheless, we speculate that the upregulation of IFNβ1 in cancer cells after treatment might be driven by the nucleic acid sensing pathway cGAS‐cGAMP‐STING, since accumulating evidence has shown that therapies that induce genomic instability, including radiation, can trigger the expression of interferons via this pathway [59, 60]. This could also explain why chemotherapeutic agents with distinct mechanisms of action such as paclitaxel and epirubicin, induce the expression of IFNβ1. Despite the fact that these drugs are known to exert their cytotoxic effect via different ways, they both induce high levels of genomic instability. Moreover, we linked IFNβ1 to the increased recovery potential observed in cancer cells in the presence of fibroblasts. Silencing of *IFNβ1* in cancer cells abrogated their recovery capacity when they were cultured with stromal fibroblasts, indicating that IFNβ1 reprograms fibroblasts into a pro‐tumourigenic state.

Inflammation is a complex process in cancer. Fibroblasts have been described as an important source of tumour‐promoting inflammation with several studies finding evidences that pro‐inflammatory fibroblasts drive malignancy and the development of metastases [44, 61, 62, 63]. In our system, this anti‐viral, pro‐inflammatory state that is induced in fibroblasts by CTX‐treated cancer cells seems to promote the recovery of cancer cells after CTX treatment. It was previously described that exposure of fibroblasts to high doses of CTX could also drive an inflammatory microenvironment [43, 64]. In contrast with these observations, in our system treatment of fibroblasts with the same chemotherapeutic doses that cancer cells were exposed to, did not drive the expression of ISGs in this cell type. In an attempt to more closely mimic the situation that is present in the tumour context, fibroblasts were grown to full confluency and the effect of CTX in their cell viability was minimal. We thus propose that differences between the experimental setups used in our as compared to previous studies could explain the differences in the results obtained. Exposure of stromal fibroblasts to IFNβ1 led to the upregulation of specific cytokines, such as *CCL5* and C‐X‐C motif chemokine ligand 10 (*CXCL10*). The impact of IFNβ1 in the expression of these two cytokines is further supported by other studies [22, 58]. *CXCL10* has been recently shown to promote a stem‐cell like phenotype in cancer cells [63]. In addition, another study has also connected pro‐inflammatory cytokines with the expansion of stem‐like cells and development of chemoresistance [43], although they implicate different cytokines and chemokines in this process. Thus, we hypothesize that activated fibroblasts by IFNβ1 may drive the expansion of populations of stem‐cell like cells that do not undergo apoptosis after the exposure to CTX. It would be interesting to investigate if the cytokines that are secreted by IFNβ1‐induced pro‐inflammatory fibroblasts are driving the expansion of these populations. Moreover, these cytokines are known immune modulators and it would be important to further explore their impact on the immune milieu in this context.

We show that IFNβ1 secreted by cancer cells drives the fibroblasts into a pro‐tumorigenic state that promotes the survival and recovery of cancer cells after CTX treatment. This state is characterized by the expression of numerous ISGs. Importantly, we find a negative correlation between *IFNB1* expression and RFS in BC patients treated with taxane and anthracycline regimens thereby attaching clinical relevance to our findings. Furthermore, disease‐free survival time in patients with high expression of the IFN signature identified in fibroblasts was significantly reduced, indicating that the activation of this signalling axis could be important in driving the appearance of relapses and can have predictive value for defining patients with higher probability of early relapses. This correlation was only observed in ER^+^ patients undergoing CTX treatment, highlighting the heterogeneity of BC. The impact of ER signalling needs to be further investigated to better understand this effect. Additionally, secretome analysis of fibroblasts is required in order to comprehend which soluble factors are responsible for promoting the survival and recovery of cancer cells after treatment with cytotoxic drugs. Ultimately, this newly identified signalling axis should be further explored as its targeting might represent a potential strategy to improve the outcome of patients to CTX treatment.

## 5 Conclusions

In summary, this study reveals a novel crosstalk between cancer cells and stromal fibroblasts that drives therapeutic failure in BC. Mechanistically, we demonstrate that upon CTX treatment, secretion of IFNβ1 drives the acquisition of a pro‐tumorigenic state in stromal fibroblasts. Considering that we further show that both IFNβ1 expression and the IFN signature identified in fibroblasts correlate with higher recurrence rates in BC, this paracrine signalling may be potentially targeted to improve the outcome of patients to CTX treatment.

## Consent for publication

All authors have read and agreed to publish this manuscript.

## Conflict of interest

The authors declare no conflict of interest.

### Peer Review

The peer review history for this article is available at https://publons.com/publon/10.1002/1878‐0261.12905.

## Author contributions

AM designed and performed the experimental work in the study. ZG performed the bioinformatic analysis of RNA‐sequencing data under the supervision of MS. Gene set enrichment analyses and patient data set analyses was done by AM. AK obtained BC patient samples, performed isolation of primary fibroblasts and contributed with critical feedback. MB‐A performed isolation of primary fibroblasts. RW generated the MCF7‐FUCCI cell line. AM and SW designed the study and wrote the manuscript. All authors read and approved the final manuscript.

## Supporting information


**Fig. S1**. Fibroblasts promote the recovery of cancer cells after high‐dose CTX.
**Fig. S2**. Cancer cells that recover are not resistant to CTX.
**Fig. S3**. Fibroblasts promote re‐entering in cell cycle of cancer cells after CTX treatment.
**Fig. S4**. MCF7 and CAF1 RNA‐sequencing.
**Fig. S5**. Fibroblasts acquire an anti‐viral state after CC with CTX‐treated cancer cells.
**Fig. S6**. Anti‐viral like state in fibroblasts is independent of nucleic acid sensing pathways.
**Fig. S7**. IFNβ1 secreted by CTX‐treated cancer cells drives fibroblasts into an anti‐viral state.
**Fig. S8**. Clinical significance of IFNβ1 axis.
**Table S1**. Chemotherapy concentration.
**Table S2**. RT‐qPCR primers and probes.
**Table S3**. List of siRNAs.
**Table S4**. Antibodies list.
**Table S5**. List of top 10 HALLMARK terms in untreated, epirubicin‐ and paclitaxel‐treated MCF7 (FDR < 0.05).
**Table S6**. Top 10 HALLMARK terms enriched in CAF1 in CC with epirubicin (CC‐E) and paclitaxel (CC‐P)‐ treated cancer cells compared to CC with untreated cancer cells.
**Table S7**. Anti‐viral (IFN) signature genes.

## Data Availability

All data generated during this study are included in this manuscript.
