# Supplementary material for: IFNβ1 secreted by breast cancer cells undergoing chemotherapy reprograms stromal fibroblasts to support tumour growth after treatment
Source: Mol Oncol. 2021 Feb 11;15(5):1308–29. doi: 10.1002/1878-0261.12905 (PMC8096792; doi:10.1002/1878-0261.12905)
Supplement: Supplementary file 1 — Fig. S1. Fibroblasts promote the recovery of cancer cells after high‐dose CTX. Fig. S2. Cancer cells that recover are not resistant to CTX. Fig. S3. Fibroblasts promote re‐entering in cell cycle of cancer cells after CTX treatment. Fig. S4. MCF7 and CAF1 RNA‐sequencing. Fig. S5. Fibroblasts acquire an anti‐viral state after CC with CTX‐treated cancer cells. Fig. S6. Anti‐viral like state in fibroblasts is independent of nucleic acid sensing pathways. Fig. S7. IFNβ1 secreted by CTX‐treated cancer cells drives fibroblasts into an anti‐viral state. Fig. S8. Clinical significance of IFNβ1 axis. Table S1. Chemotherapy concentration. Table S2. RT‐qPCR primers and probes. Table S3. List of siRNAs. Table S4. Antibodies list. Table S5. List of top 10 HALLMARK terms in untreated, epirubicin‐ and paclitaxel‐treated MCF7 (FDR < 0.05). Table S6. Top 10 HALLMARK terms enriched in CAF1 in CC with epirubicin (CC‐E) and paclitaxel (CC‐P)‐ treated cancer cells compared to CC with untreated cancer cells. Table S7. Anti‐viral (IFN) signature genes. [file MOL2-15-1308-s001.pdf]

Supplementary Information

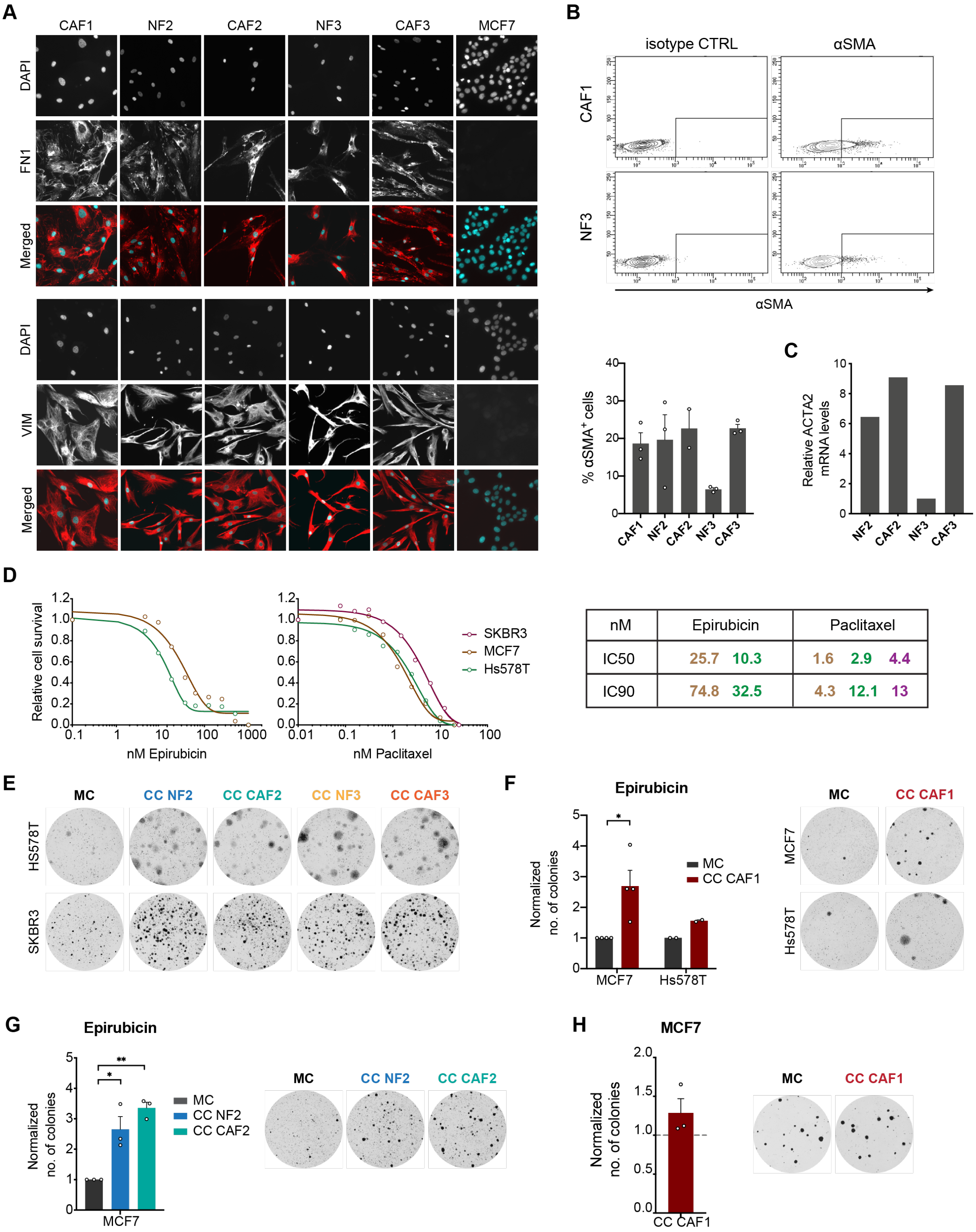

nM	Epirubicin	Paclitaxel
IC50	25.7 10.3	1.6 2.9 4.4
IC90	74.8 32.5	4.3 12.1 13

Supplementary Figure 1. Fibroblasts promote the recovery of cancer cells after high-dose chemotherapy.

A: Representative images of Fibronectin and Vimentin expression in primary fibroblasts and in MCF7 determined by immunofluorescence.

B-C: Quantification of αSMA expression in the different fibroblast lines using flow cytometry (FC) analysis (**B**) and RT-qPCR (**C**). For FC analysis, positive cells were determined based on isotype control levels (See representative FC plot on top panel of B). Each dot represents an independent replicate (For all fibroblasts, except CAF2: n=2). C shows ACTA2 mRNA levels in the several fibroblasts determined by RT-qPCR (n=1).

D: Drug response curves of MCF7, Hs578T and SKBR3 to epirubicin and paclitaxel determined by nuclei count. Cancer cells were exposed for three days to chemotherapeutic agents after which they were imaged and analysed. Results from three biological replicates (n=3) are shown as mean without variation for visual clarity (left panel). IC50 and IC90 values based on three day drug exposure were calculated in GraphPad Prism 8.0 for the different cell lines and are shown in the right panel.

E: Representative images of the colony formation assay of Hs578T and SKBR3 exposed to 8 nM paclitaxel in MC and CC with fibroblast pair #2 and #3 (n=1).

F: Quantification of MCF7 and Hs578T colonies in MC and CC with CAF1 at the end of recovery after treatment with epirubicin. Each dot represents an independent replicate (MCF7: n=6; Hs578T: n=2). Representative pictures of colony formation assay wells at end-point are shown bellow.

G: Recovery assay of MCF7 exposed to 70 nM epirubicin in MC or CC with CAF2 or NF2. Quantification of the number of colonies after 15 days of recovery for three independent experiments (n=3) is shown in top panel and representative pictures in lower panel.

H: Colony formation assay of untreated MCF7 in CC with CAF1. Quantification is normalized to number of colonies of untreated MCF7 in MC. Representative images from three independent experiments (n=3) are shown on the lower panel.

For all recovery assays shown, cancer cells were treated for three days with chemotherapy and then allowed to recover in MC and CC for 15 days, at which point the colonies in the wells were imaged and quantified.

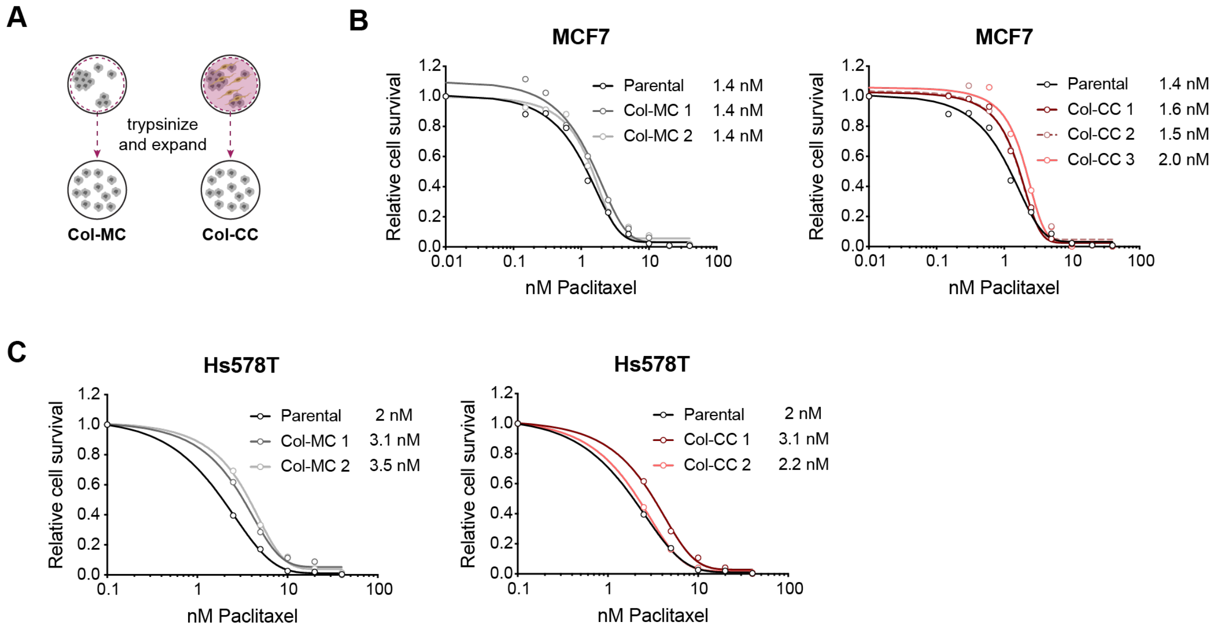

Supplementary Figure 2. Cancer cells that recover are not resistant to chemotherapy.

A: Schematic layout of colony analysis. Colonies from MC (Col-MC) and CC (Col-CC) were collected and expanded for drug response assay.

B-C: Drug response curves to paclitaxel treatment for three days of MCF7 (**B**) and Hs578T (**C**) colonies determined by nuclei count. Results are shown as mean without variation for visual clarity. For MCF7, colonies from two and three independent experiments were collected for Col-MC (n=2) and Col-CC (n=3), respectively. For Hs578T, colonies from two experiments for each condition were collected and used for the analysis (n=2). IC50 values were calculated in GraphPad Prism 8.0 for both cell lines.

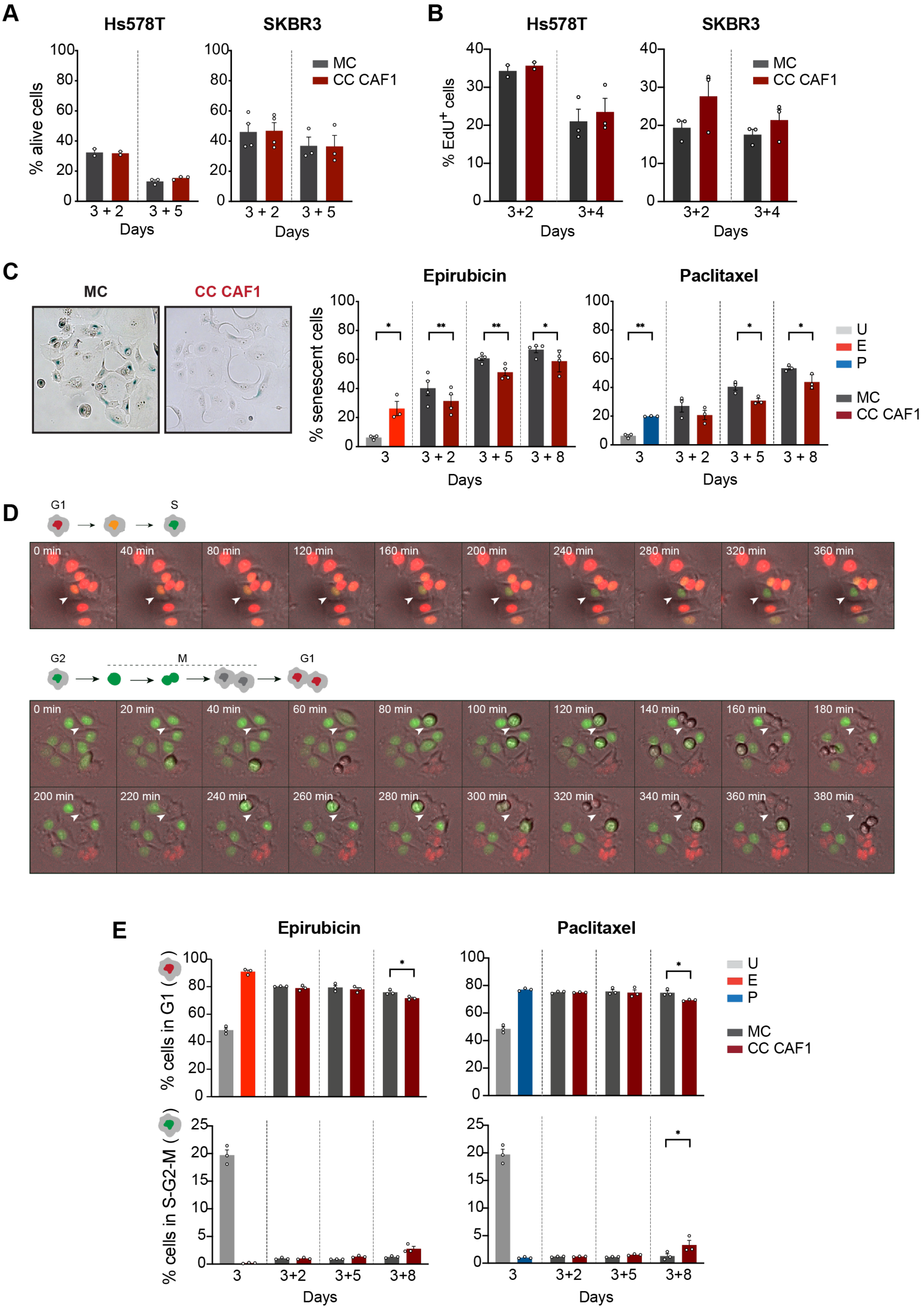

Supplementary Figure 3. Fibroblasts promote re-entering in cell cycle of cancer cells after chemotherapy treatment.

A: Quantification of DAPI negative cells in Hs578T and SKBR3 for both MC and CC conditions after two (3+2) (n=2 for Hs578T and n=4 for SKBR3) and five (3+5) (n=3 for both cell lines) days of recovery was done. Graphs show the percentage of alive cells (%) calculated by dividing the number of DAPI negative cells by the total number of singlets. Each dot represents an independent biological replicate.

B: Ratio between the number of positive cells for EdU (EdU^+) and total number of cells counted by DAPI staining was done to calculate the percentage (%) of EdU^+ cells in Hs578T (n=2 for day 3+2, n=3 for day 3+4) and SKBR3 (n=3) after the cytotoxic stimuli with chemotherapy.

C: SA-β-Galactosidase staining of MCF7 (n=4 for epirubicin, except for day 3 where n=3; n=3 for paclitaxel). Left panel shows a representative picture of cells in MC and CC with fibroblasts. Quantification of the % of senescent cells from n≥3 is shown in the right panel. Data is shown as mean ± SEM and each dot represents an independent biological replicate. P values were calculated using unpaired two-tailed t-test on biological replicates. * p<0.05, ** p<0.01.

D: Images from a time-lapse experiment of MCF7-FUCCI showing the different cell cycle phases transitions. White arrow shows transition from G1 to S phase (upper panel) and G2-M-G1 transitions (lower panel). Time stamp is shown in upper left corner of each picture.

E: Quantification of Cdt1-RFP (upper panel) and Geminin-GFP (lower panel) expressing cells in MCF7-FUCCI by flow cytometry after treatment with epirubicin or paclitaxel at several recovery points. Data is shown as mean ± SEM from three independent experiments (n=3). P values were calculated using unpaired two-tailed t-test on biological replicates. * p<0.05.

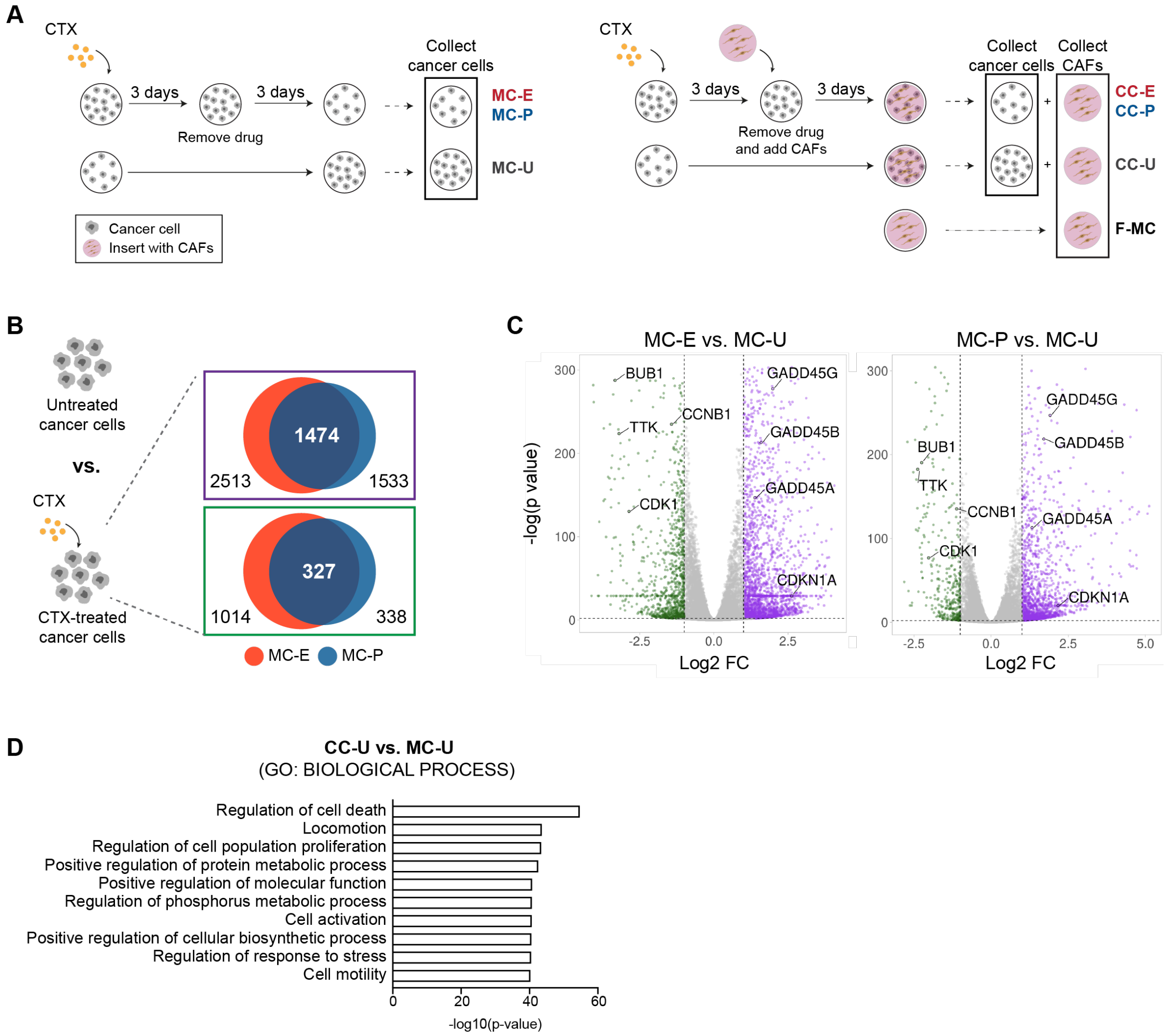

Supplementary Figure 4. MCF7 and CAF1 RNA-sequencing.

A: RNA-sequencing schematic layout. MCF7 treated with either epirubicin (E) or paclitaxel (P) were allowed to recover for three days in the absence (MC) or presence of fibroblasts (CC). At time point day 3+3, both MCF7 and CAF1 were collected and their gene expression profiles assessed by RNA-sequencing. Three replicates (n=3) per condition were sequenced.

B: Venn diagram depicting differently upregulated (FC>2) (purple box) and downregulated (FC<0.5) (green box) genes in MCF7 after chemotherapy (CTX) treatment compared to untreated cells. Red circle represents the differently expressed genes after epirubicin treatment (MC-E) while the blue circle shows the ones in paclitaxel treatment (MC-P).

C: Volcano plots showing the significantly differently expressed genes in CTX-treated MCF7 compared to untreated (MC-U). Upregulated genes are shown in purple and downregulated

in green. Example of genes related to the p53 pathway (upregulated) and cell cycle progression (downregulated) are highlighted. Volcano plots were designed using the *VolcaNose*[31] tool.

D: Analysis of data generated by RNA-sequencing using GSEA (GO: Biological process). Main pathways (top 10) upregulated in untreated MCF7 cells in co-culture with CAF1 compared to mono-culture are shown.

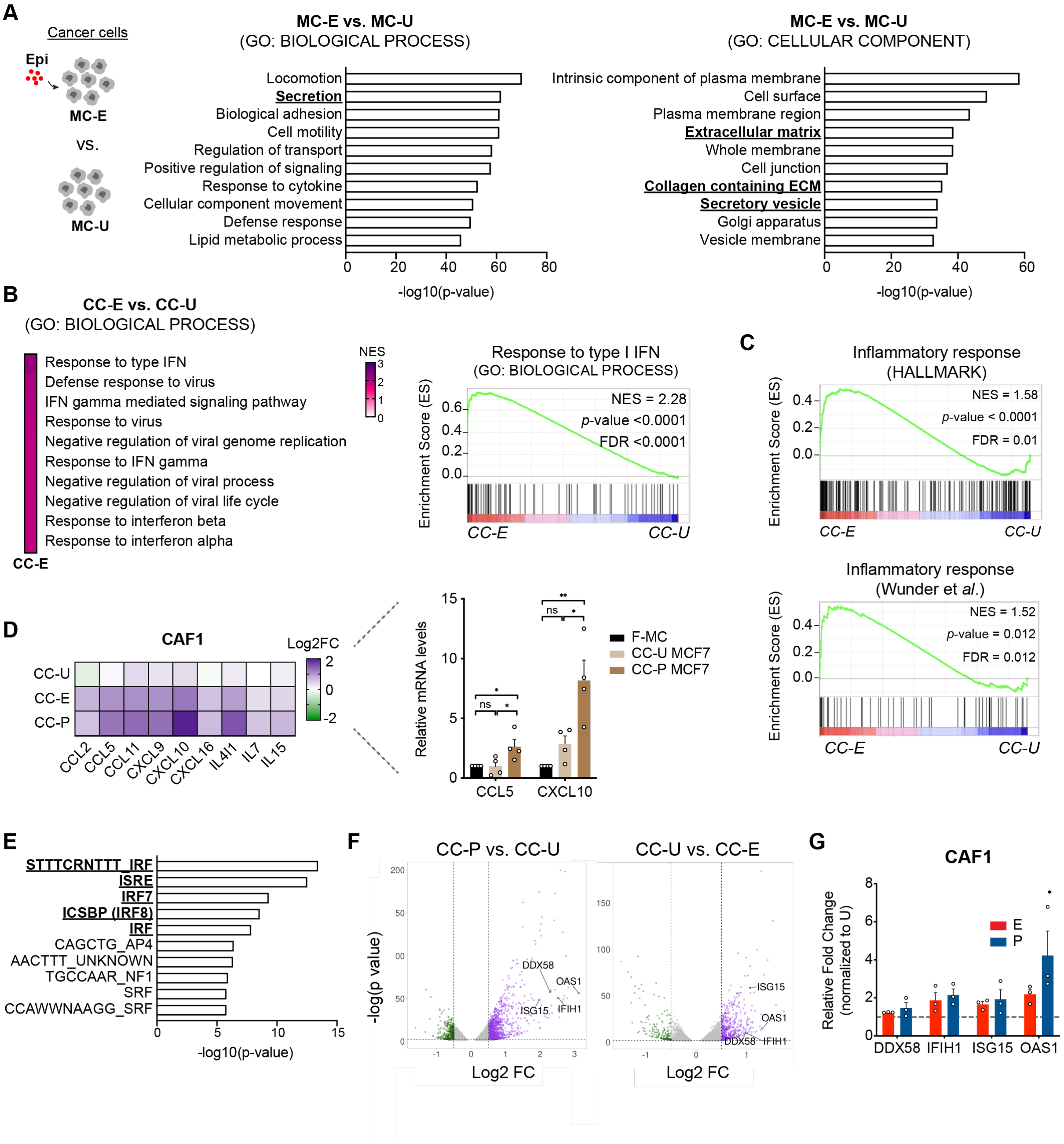

Supplementary Figure 5. Fibroblasts acquire an anti-viral state after co-culture with chemotherapy-treated cancer cells.

A: Top 10 enriched GO terms for biological process (left panel) and cellular component (right panel) of top 2000 upregulated genes in epirubicin-treated MCF7 (MC-E) compared to untreated (MC-U). Terms related to secreted factors are highlighted in bold.

B: GSEA analysis of CAF1 in CC-E compared to CC-U. Top 10 significantly enriched GO:BP terms enriched in CC-E are shown. NES = normalized enriched score. Enrichment curve for the top biological process (Response to type I interferon) in CAF1 in CC-E versus CC-U is

shown in right panel. FDR = false discovery rate. P values were calculated by random permutation tests.

C: CAF1 in co-culture with epirubicin-treated cancer cells (CC-E) acquire an inflammatory signature. Enrichment analysis revealed in the top 10 hallmark signatures (HALLMARK), the inflammatory response gene set (upper panel). An independent gene set for inflammation (Wunder *et al.*[32]) was used for gene set enrichment analysis (lower panel).

D: Heat-map with the expression of several pro-inflammatory cytokines in CAF1 in co-culture with untreated MCF7 (CC-U) and with epirubicin (CC-E) or paclitaxel-treated MCF7 (CC-P). Relative expression is normalised to CAF1 in mono-culture and is shown as log2 fold change. Right panel shows RT-qPCR analysis of the expression of *CCL5* and *CXCL10* in CAF1 grown for 5 days in mono-culture (F-MC) or co-culture with untreated (CC-U) or paclitaxel-treated (CC-P) MCF7. Values were normalized to CAF1 in F-MC. mRNA levels were normalized against two house-keeping genes (*ACTB* and *PUM1*). Each dot represents an independent experiment (n=4). P values were calculated using one-way ANOVA in biological replicates. * p<0.05, ** p<0.01.

E: Top 10 over-represented transcription factors and motifs involved in the regulation of genes upregulated in CAF1 in CC-E. Transcription factors known to be involved in anti-viral response are underlined and highlighted in bold.

F: Volcano plots of differently expressed genes in CAF1 in CC-P (left panel) or CC-E (right panel) compared to CC-U. Upregulated genes are shown in purple and downregulated genes in green. Anti-viral genes *DDX58*, *IFIH1*, *ISG15* and *OAS1* are highlighted. Volcano plot was designed using *VolcaNose*[31] tool.

G: Anti-viral genes expression in CAF1 after treatment with 70 nM epirubicin (E) or 4 nM paclitaxel (P). Expression level was analysed using qRT-PCR and values normalized to untreated (U) CAF1. P value was calculated using one-way ANOVA, * p<0.05. Data is shown as mean ± SEM from three independent experiments (n=3).

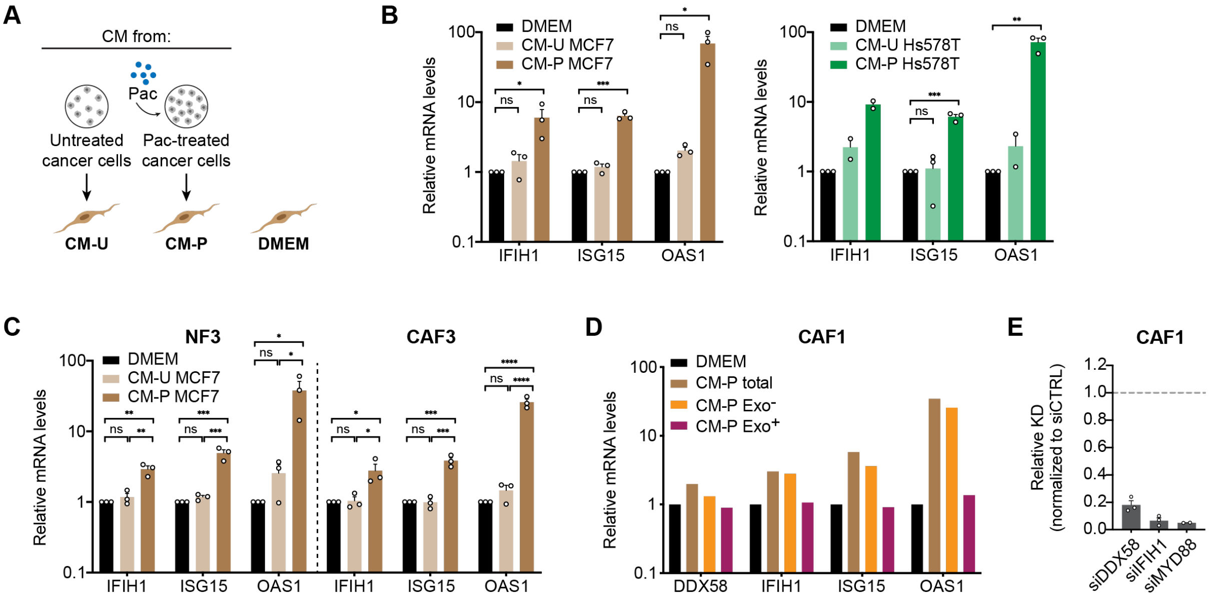

Supplementary Figure 6. Anti-viral like state in fibroblasts is independent of nucleic acid sensing pathways.

A: Schematic layout for conditioned-media experiment. Supernatant from untreated (CM-U) or paclitaxel-treated (CM-P) cancer cells was collected and added to primary fibroblasts. Fibroblasts grown in media are referred to as DMEM.

B: Impact of MCF7 (left) (n=3) and Hs578T (right) (n=3, except for *IFIH1* where n=2) conditioned-media in the expression of *IFIH1*, *ISG15* and *OAS1* in CAF1 determined by RT-qPCR.

C: RT-qPCR analysis of *IFIH1*, *ISG15* and *OAS1* expression in fibroblast pair #3 (NF3 and CAF3) (n=3).

In **B** and **C** values were normalized to CAF1 grown in DMEM. mRNA levels were normalized against two house-keeping genes (*ACTB* and *PUM1*). Data is shown as mean ± SEM from at least two independent experiments (n≥2). Each dot represents a biological replicate. P values were calculated using one-way ANOVA. * p<0.05, ** p<0.01, *** p<0.001, **** p<0.0001.

D: Impact of exosomes in the anti-viral state of CAF1. Conditioned-media from paclitaxel-treated MCF7 was added directly to CAF1 (CM-P total) or ultra-centrifuged before. Supernatant fraction – exosome depleted (CM-P Exo^-) – and pellet fraction – exosome enriched (CM-P Exo^+) – were separated and added to CAF1. Expression of *DDX58*, *IFIH1*,

ISG15 and *OAS1* was assessed using RT-qPCR. Values were normalized to CAF1 grown in DMEM. mRNA levels were normalized against two house-keeping genes (*ACTB* and *PUM1*). Data from one experiment (n=1) is shown.

E: Knock-down efficiency determined RT-qPCR of the RIG-I pathway cytosolic receptors – *DDX58* and *IFIH1* (n=3) – and of the TOLL-like receptor adaptor protein – *MYD88* (n=2). Values were normalized to expression in cells transfected with a control siRNA (siCTRL).

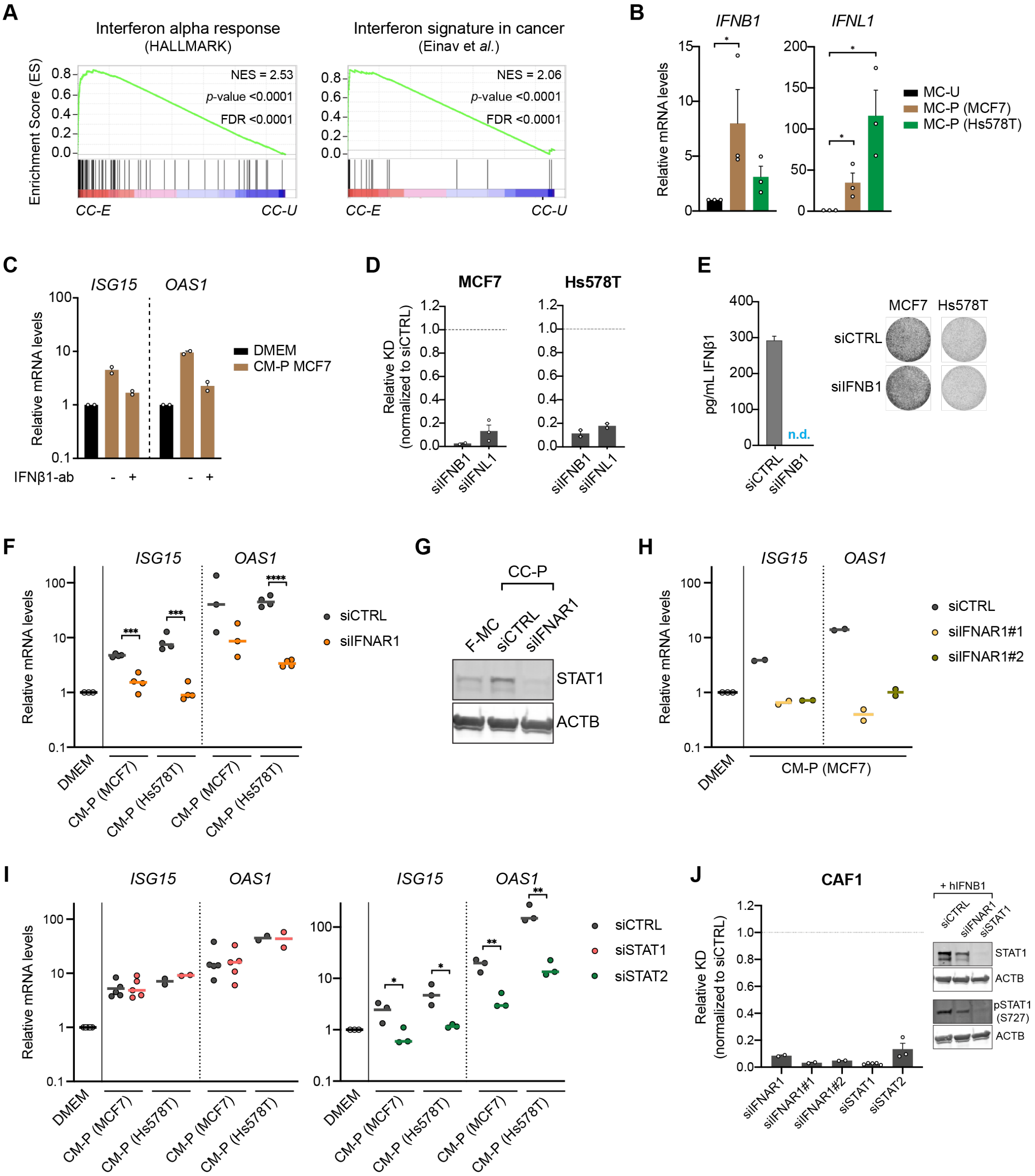

Supplementary Figure 7. IFNβ1 secreted by chemotherapy-treated cancer cells drives fibroblasts into an anti-viral state.

A: GSEA (HALLMARK) revealed interferon alpha response as number one enriched gene set in fibroblasts in co-culture with epirubicin-treated cancer cells (CC-E) compared with untreated cancer cells (CC-U) (left panel). Independent gene set for interferon signature[36] was used for enrichment analysis (right panel). NES = normalized enrichment score, FDR = false discovery rate. P values were determined by random permutation tests.

B: RT-qPCR analysis of the expression of *IFNB1* and *IFNL1* in MCF7 and Hs578T treated with paclitaxel (MC-P). Values were normalized to untreated cancer cells (MC-U). mRNA levels were normalized against two house-keeping genes (*ACTB* and *PUM1*). Each dot represents an independent experiment (n=3). P values were calculated using unpaired two-tailed t-test in biological replicates. * p<0.05.

C: Impact of neutralizing antibody against IFNβ1 (IFNβ1-ab) in anti-viral gene expression in CAF1 measured by qRT-PCR. *ISG15* and *OAS1* expression in CM-P conditions were normalized to DMEM. Data from two independent experiments (n=2) is shown.

D-E: KD efficiency of siIFNB1 and siIFNL1 in MCF7 (n=3) and Hs578T (n=2), measured by RT-qPCR (**D**). **E**: KD efficiency of siIFNB1 in MCF7, measured by ELISA (n=2). Gene expression values were normalized to cells transfected with a control siRNA (siCTRL). Pictures show representative wells of MCF7 and Hs578T transfected either with siCTRL or siIFNB1 after six days of transfection.

F: qRT-PCR of anti-viral genes – *ISG15* and *OAS1* – in CAF1 transfected with an siCTRL or siIFNAR1 (pool) and exposed to the supernatant of paclitaxel-treated MCF7 (n=4 and n=3 for *ISG15* and *OAS1*, respectively) or Hs578T (n=4). Values were normalized to CAF1 grown in media (DMEM). Each dot represents an independent biological replicate. P values were calculated using one-way ANOVA. ** p<0.01, *** p<0.001, **** p<0.0001.

G: Immunoblot of STAT1 in CAF1 in mono-culture (F-MC) or in co-culture with paclitaxel-treated MCF7 (CC-P) and transfected with a control siRNA (siCTRL) or the siIFNAR1 (pool). ACTB was used as a loading control.

H: Two independent siRNAs against siIFNAR1 were used and their impact in *ISG15* and *OAS1* measured by RT-qPCR. Data is from two independent experiments (n=2).

I: Impact of siSTAT1 (left panel) (n=5 for MCF7, n=2 for Hs578T) and siSTAT2 (right panel) (n=3) in anti-viral gene expression. CAF1 were exposed to the supernatant of either paclitaxel-treated MCF7 or Hs578T. Values were normalized to CAF1 grown in media (DMEM). Each dot represents an independent biological replicate. P values were calculated using one-way ANOVA. * p<0.05, ** p<0.01.

J: Left panel shows KD efficiency for siRNAs measured by RT-qPCR. Values were normalized to cells transfected with a control siRNA (siCTRL) (siIFNAR1s n=2, siSTAT1 n=5, siSTAT2 n=3). Data is shown as mean ± SEM. Each dot represents an independent replicate. Immunoblot for STAT1 and pSTAT1 (S727) for lysates of cells transfected with either siCTRL, siIFNAR1 or siSTAT1 is shown on the right panel.

In all RT-qPCR experiments, mRNA levels were normalized against two house-keeping genes (*ACTB* and *PUM1*).

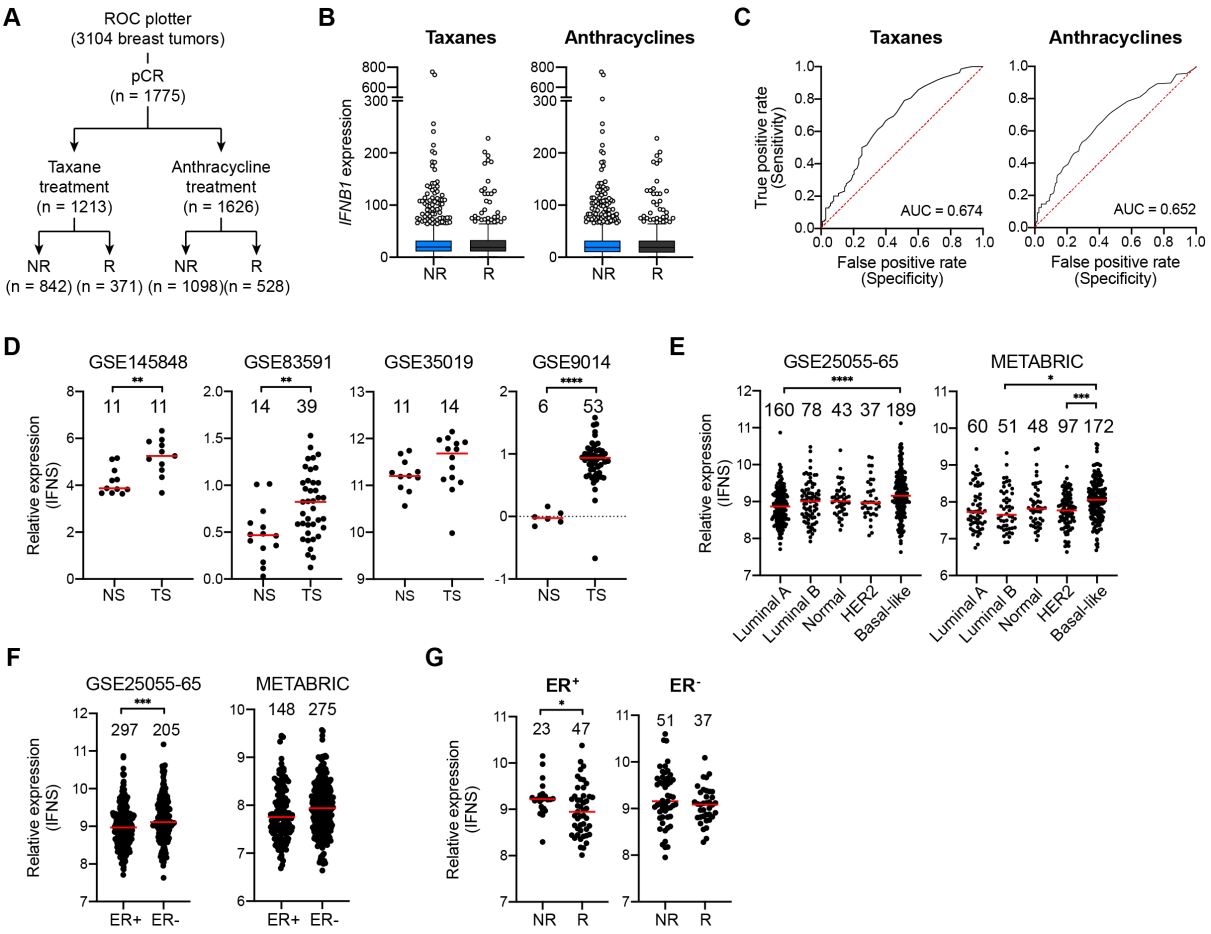

Supplementary Figure 8. Clinical significance of IFNβ1 axis.

A: Schematic overview of patient number for each group in ROC plotter – pathological complete response (pCR).

B: Analysis of *IFNB1* expression and pCR using ROC plotter in breast cancer patients treated with taxanes (left) or anthracyclines (right).

NR = non-responder, R = responder. Response is determined based the presence of tumour cells after treatment. Patients with tumour cells present after treatment are classified as non-responders, while patients with no tumour cells are called responders.

C: Area under the curve for correlation analysis between *IFNB1* expression and RFS.

D: Analysis in LCM datasets – GSE145848, GSE8391, GSE35019 and GSE9014 – of the IFNS expression in normal stroma (NS) and tumour stroma (TS). Each dot represents one sample. Patient matched data was available in GSE145848. P values were then calculated using two-tailed paired t-test. ** p<0.01. For the other datasets, no matched data was available

and p values were calculated using two-tailed unpaired t-test. * $p<0.05$, **** $p<0.0001$. Red line represents median expression in each group. Numbers show total samples analysed in each group.

E: Relative expression of the IFN signature (IFN) in patients divided by PAM50 subtypes in GSE25055-GSE25065 and METABRIC datasets. Each dot represents one patient. P values were calculated using one-way ANOVA. * $p<0.05$, *** $p<0.001$, **** $p<0.0001$. Red line represents median expression in each group. Numbers show total samples analysed in each group.

F: Relative expression of the IFN signature (IFN) in ER-positive (ER^+, n=148) and -negative (ER^-, n=275) patients from METABRIC dataset. Each dot represents one patient. P values were calculated using two-tailed unpaired t-test. Red line represents median expression in each group.

Supplementary Table 1. Chemotherapy concentration.

Cell line	Epirubicin (nM)	Paclitaxel (nM)
**HS578T**	70	8
**MCF7**	70	4
**SKBR3**	-	8

Supplementary Table 2. RT-qPCR primers and probes.

Gene	Primers sequence	Probe
**ACTA2**	GCACTGCCTTGGTGTGTG<br>TCCCATTCCCACCATCAC	21
**ACTB**	CCAACCGCGAGAAGATGA<br>CCAGAGGCGTACAGGGATAG	64
**CCL5**	ACAGGTCAAGGATGCCAAAG<br>GTTCTTTCGGGTGACAAAGC	56
**CXCL10**	AAGCAGTTAGCAAGGAAAGGTC<br>GACATATACTCCATGTAGGGAAGTGA	34
**DDX58**	ATGTGGGCAATGTCATCAAA<br>AAGCACTTGCTACCTCTTGCTC	13
**IFIH1**	TTTTGCAGATTCTTCTGTAGTTTCA<br>TGCTGTTATGTCCAAGACTTTCA	29
**IFNAR1**	TGACCAGAAATGAACTGTGTCAA<br>GACCTCAGGCTCCCAGTGTA	18
**IFNB1**	CTTTGCTATTTTCAGACAAGATTCA<br>GCCAGGAGGTTCTCAACAAT	20
**IFNL1**	GGCCTGTATCCAGCCTCA<br>AAGGTGACAGATGCCTCCAG	79
**ISG15**	GAGGCAGCGAACTCATCTTT<br>AGCATCTTCACCGTCAGGTC	76
**MYD88**	GTCCCACGGACAGCAGAG<br>GCAGATGAAGGCATCGAAA	9
**OAS1**	GGTGGAGTTCGATGTGCTG<br>AGGTTTATAGCCGCCAGTCA	37
**PUM1**	TCACATGGATCCTCTTCAAGC<br>CCTGGAGCAGCAGAGATGTAT	86

Supplementary Table 3. List of siRNAs.

Gene	Catalogue number	Target sequence
**ON-TARGET (CTRL)**	D-001810-10-20	1 – UGGUUUACAUGUCGACUAA<br>2 – UGGUUUACAUGUUGUGUGA<br>3 – UGGUUUACAUGUUUUCUGA<br>4 – UGGUUUACAUGUUUUCCUA
**DDX58**	J-012511-05	1 – GCACAGAAGUGUAUAUUGG<br>2 – CCACCACACUAGUAAACAA<br>3 – CGGAUUAGCGACAAAUUUA<br>4 – UCGAUGAGAUUGAGCAAGA
**IFIH1**	J-013041-05	1 – GAAUAACCCAUCACUAAUA<br>2 – GCACGAGGAAUAAUCUUUA<br>3 – UGACACAAUUCGAAUGAUA<br>4 – CAAUGAGGCCCUACAAAUU
**MYD88**	J-004769-05	1 – CGACUGAAGUUGUGUGUGU<br>2 – GCUAGUGAGCUCAUCGAAA<br>3 – GCAUAUGCCUGAGCGUUUC<br>4 – GCACCUGUGUCUGGUCUAU
**IFNB1**	J-019656-05	1 – GGAAUGAGACUAUUGUUGA<br>2 – AUGGGAGGAUUCUGCAUUA<br>3 – CAACUUGCUUGGAUUCCUA<br>4 – GCAUUGACCAUCUAUGAGA
**IFNL1**	J-007982-05	1 – UCAAAUAUGUGGCCGAUGG<br>2 – GAACGUCAACCCACCCUGA<br>3 – CCGUGGUGCUGGUGACUUU<br>4 – CCACAUUGGCAGCUUCAAA
**IFNAR1**	J-020209-05	1 – GCGAAAGUCUUCUUGAGAU<br>2 – UGAAACCACUGACUGUAUA<br>3 – GAAAAUUGGUGUCUAUAGU<br>4 – GAAGAUAAGGCAAUAGUGA

Supplementary Table 4. Antibodies list.

Antibody	Catalogue number	Company	Working concentration/ Application
**Actin B**	8691002	MP Biomedicals	1:10000 / WB
**pSTAT1 (S727)**	9177	CST	1:1000 / WB
**STAT1**	9172	CST	1:1000 / WB

Supplementary Table 5. List of top 10 HALLMARK terms in untreated, epirubicin- and paclitaxel-treated MCF7 (FDR<0.05)

- MC-E vs. CC-E (MCF7) – Enriched in CC

Name	NES	FDR	NOM p-value
**HALLMARK E2F TARGETS**	**1.96**	**0**	**0**
**HALLMARK G2M CHECKPOINT**	1.90	0	0
**HALLMARK MYC TARGETS V1**	1.77	0.001	0
**HALLMARK PROTEIN SECRETION**	1.59	0.010	0.002
**HALLMARK ANDROGEN RESPONSE**	1.59	0.027	0.005
**HALLMARK SPERMATOGENESIS**	1.41	0.037	0.011

- MC-P vs. CC-P (MCF7) – Enriched in CC

Name	NES	FDR	NOM p-value
**HALLMARK MYC TARGETS V1**	2.05	0	0
**HALLMARK E2F TARGETS**	**2.02**	**0**	**0**
**HALLMARK G2M CHECKPOINTS**	1.91	0	0
**HALLMARK PROTEIN SECRETION**	1.82	0	0
**HALLMARK MTORC1 SIGNALING**	1.60	0.005	0
**HALLMARK SPERMATOGENESIS**	1.49	0.0130	0.004
**HALLMARK ANDROGEN RESPONSE**	1.43	0.026	0.025

- MC-U vs. CC-U (MCF7) – Enriched in CC

Name	NES	FDR	NOM p-value
**HALLMARK TNFA SIGNALING VIA NFKB**	2.60	0	0
**HALLMARK INFLAMMATORY RESPONSE**	2.15	0	0
**HALLMARK MYC TARGETS V1**	2.12	0	0
**HALLMARK EMT TRANSITION**	2.01	0	0

**HALLMARK UNFOLDED PROTEIN RESPONSE**	2.00	0	0
**HALLMARK MTORC1 SIGNALING**	2.00	0	0
**HALLMARK ESTROGEN RESPONSE EARLY**	1.98	0	0
**HALLMARK IL2 STAT5 SIGNALING**	1.97	0	0
**HALLMARK ALLOGRAFT REJECTION**	1.92	0	0
**HALLMARK G2M CHECKPOINT**	1.91	0	0

Supplementary Table 6. Top 10 HALLMARK terms enriched in CAF1 in co-culture with epirubicin (CC-E) and paclitaxel (CC-P)- treated cancer cells compared to co-culture with untreated cancer cells.

- CC-U vs. CC-E (CAF1) – Enriched in CC

Name	NES	FDR	NOM p-value
**HALLMARK INTERFERON ALPHA RESPONSE**	2.53	0	0
**HALLMARK INTERFERON GAMMA RESPONSE**	2.39	0	0
**HALLMARK TNFA SIGNALING VIA NKFB**	2.11	0	0
**HALLMARK UV RESPONSE DN**	1.90	0	0
**HALLMARK IL6 JAK STAT3 SIGNALING**	1.80	0	0
**HALLMARK KRAS SIGNALING UP**	1.76	0.001	0
**HALLMARK APOPTOSIS**	1.73	0.001	0
**HALLMARK INFLAMMATORY RESPONSE**	**1.58**	**0.011**	**0**
**HALLMARK ESTROGEN RESPONSE EARLY**	1.58	0.01	0.001
**HALLMARK HYPOXIA**	1.58	0.009	0

- CC-U vs. CC-P (CAF1) – Enriched in CC

Name	NES	FDR	NOM p-value
**HALLMARK INTERFERON ALPHA RESPONSE**	2.83	0	0
**HALLMARK INTERFERON GAMMA RESPONSE**	2.81	0	0
**HALLMARK TNFA SIGNALING VIA NKFB**	2.35	0	0
**HALLMARK UV RESPONSE DN**	2.01	0	0
**HALLMARK IL6 JAK STAT3 SIGNALING**	1.91	0	0
**HALLMARK MITOTIC SPINDLE**	1.86	0	0
**HALLMARK INFLAMMATORY RESPONSE**	**1.86**	**0**	**0**
**HALLMARK TGF BETA SIGNALING**	1.81	0	0
**HALLMARK KRAS SIGNALING UP**	1.69	0.002	0
**HALLMARK IL2 STAT5 SIGNALING**	1.66	0.003	0

Supplementary Table 7. Anti-viral (IFN) signature genes.

Gene	Gene	Gene
**BST2**	IFI6	CYP27B1
**IFITM1**	IKBKE	JUN
**IFITM3**	USP18	JUNB
**ISG15**	EGR1	CSF2RB
**RSAD2**	XAF1	HMOX1
**IFIT1**	IFI35	F3
**OAS1**	GBP1	CEBPD
**OAS2**	PLSCR1	IRS2
**OAS3**	DDX58	KLF5
**MX1**	IFIH1	BCLAF1
**STAT1**	DHX58	DUSP1
**SAMHD1**	DDX60	GCLM
**IFI27**	IFI44L	GCLC
**IRF7**	GATA3	TLE4
**IFIT2**	IFI44	TRIM14
**IFIT3**	CCL2	CLEC4A
**MX2**	NR1D1	APOL1
		HERC6
